# Effects of Paraffin/Sisal Composite Phase Change Fibers on the Rheological and Heat-Transfer Behaviors of 3D-Printed Concrete

**DOI:** 10.3390/ma19204326

**Published:** 2026-10-14

**Authors:** Chun Chen, Size Hua, Wenqin Xu, Qi Fu

**Affiliations:** State Key Laboratory of Engineering Materials for Major Infrastructure, School of Material Science and Engineering, Southeast University, Nanjing 211189, China; 220242767@seu.edu.cn (S.H.); m13395138628@163.com (W.X.); 17775019206@163.com (Q.F.)

**Keywords:** phase change fibers, 3D-printed concrete, rheological properties, heat-transfer behavior, pore distribution

## Abstract

This study incorporated self-prepared paraffin/sisal composite phase change fibers (PCFs) into three-dimensional printed concrete (3DPC) to enhance its thermal properties. The effects of fiber type and dosage on the rheological and thermal properties of 3DPC were systematically investigated. The mechanisms underlying these effects were examined in terms of the internal pore distribution and fiber dispersion. The results indicate that differences in fiber dispersion, which are likely governed by fiber surface characteristics, are closely associated with the pore distribution within the 3DPC and, consequently, with its rheological and thermal properties. Compared with sisal-fiber-reinforced and fiber-free 3DPC, PCF-containing 3DPC exhibited lower thermal conductivity and a slower surface temperature rise under the tested conditions, and its heat-transfer behavior differed among the X, Y, and Z directions. These findings suggest that the incorporation of PCFs can enhance the thermal insulation of 3DPC under the tested conditions.

## 1. Introduction

Three-dimensional (3D)-printed concrete (3DPC) is a sustainable construction method that reduces labor and time costs, minimizes the use of formwork, and decreases construction waste while increasing the uniqueness and flexibility of architectural designs [1,2]. To create and maintain a comfortable living and working environment, buildings rely on heating, ventilation, and air conditioning (HVAC) systems for environmental control throughout their service life. Indoor temperature regulation, including heating, cooling, and ventilation, accounts for up to 40% of global energy consumption and 30% of annual global greenhouse gas emissions [3]. Reducing building energy consumption has therefore long been a critical research focus.

Similar to traditional cast-in-place concrete structures, 3DPC buildings require adequate thermal insulation to achieve an acceptable indoor thermal environment, which is essential for occupant health [4,5]. Current strategies for improving the thermal insulation performance of 3DPC fall into three categories: (i) structural optimization, in which the design flexibility of 3D printing is used to produce walls with different cross-sectional geometries, and the effects of cavity shape and infill pattern on thermal performance are examined experimentally or numerically [6,7]; (ii) 3D-printed foamed concrete, whose cellular porous structure limits heat transfer, although its thermal and mechanical properties depend on density and pore structure [8,9]; and (iii) the incorporation of other low-thermal-conductivity materials, such as externally attached insulation panels or insulating mortar, or the filling of printed cavities with insulating materials [5]. Low-conductivity constituents such as aerogel have also been added directly to printable mixtures [10]. Most of these measures, however, require additional materials or processing steps, which increases the complexity of construction.

Extrusion-based printing imposes specific rheological requirements on cementitious materials, because the fresh paste must be extruded continuously and then retain its shape after deposition [11]. Fiber reinforcement has therefore been introduced into printable cementitious materials to improve their mechanical performance. Pham et al. [12] showed that the length, dosage, and alignment of steel fibers govern the mechanical performance and anisotropy of 3DPC. Hambach and Volkmer [13] investigated printed cement pastes containing carbon, glass, or basalt fibers and observed preferential fiber alignment along the printing path. Soltan and Li [14] developed printable polymer-fiber-reinforced cementitious composites by tailoring the fresh-state rheology while retaining tensile strain-hardening behavior. Xiao et al. [15] further examined the mechanical and microstructural evolution of printed concrete containing polyethylene fibers and recycled sand at elevated temperatures. For natural-fiber cement pastes, Galicia-Aldama et al. [16] demonstrated that fiber addition modified the rheological behavior. Because these fibers exhibit a lower thermal conductivity than the cement matrix, their dosage and spatial arrangement also affect the effective thermal conductivity of the composite [17,18]. In fiber-reinforced 3DPC, fiber dosage has been reported to increase both the static and dynamic yield stresses while reducing the plastic viscosity, and fibers tend to align along the printing direction, which contributes to the anisotropic thermal and mechanical performance of the printed material. Natural plant fibers are of particular interest in this context, because their hollow, interconnected lumens and rough surfaces provide internal space for the stable storage of phase change materials and thus make them attractive low-cost carriers [19]. These considerations indicate that the type and dosage of fibers influence the rheological behavior of 3DPC and, through the resulting pore structure and fiber arrangement, its heat-transfer behavior as well.

Each of the strategies mentioned above for enhancing the thermal performance of 3DPC has inherent limitations. Phase change materials (PCMs) absorb and release latent heat at specific temperatures or within a certain temperature range, thereby providing latent thermal storage that complements conventional insulation [20,21], and their incorporation into cementitious materials as functional fillers or aggregate replacements has been widely investigated through microencapsulation and porous carriers. Jayalath et al. [20] reported that replacing part of the fine aggregate with microencapsulated PCMs increased the heat capacity and reduced the thermal conductivity, but also decreased the compressive strength; Hunger et al. [21] observed comparable thermal and mechanical effects in self-compacting concrete. Other approaches include PCM encapsulation using cenospheres [22] and the incorporation of PCMs into expanded-clay mortar for indoor temperature regulation [23]. Studies on encapsulated PCMs in cement mortars [24] and on their effect on mortar rheology [25] further indicate that the fresh-state and hardened properties of PCM-containing composites must be considered together. Šavija and Schlangen [26] used PCMs to mitigate early-age thermal cracking. Research on PCM-containing cementitious materials has thus addressed both early-age thermal control and thermal storage for building applications.

The incorporation of PCMs has also been explored specifically for 3DPC. Khan et al. [27] discussed multifunctional printed concrete, including its potential for thermal regulation. Shahzad et al. [28] investigated the influence of PCMs on the behavior of concrete for construction 3D printing, including its viscosity, flowability, and setting time. Cui et al. [29] studied the printability and thermal properties of mixtures containing microencapsulated paraffin and demonstrated temperature regulation in printed room models. Brooks et al. [30] evaluated the printability, microstructure, mechanical properties, and thermal behavior of such mixtures and showed that the effects of microencapsulated PCMs depended on the dosage. Hao et al. [31] investigated the thermal conductivity of 3DPC containing recycled fine aggregates loaded with PCMs. Christen et al. examined PCM-impregnated recycled brick aggregates in printable mixtures [32] and subsequently assessed the resulting thermal performance through experiments and validated simulations [33,34]. These studies provide evidence for thermal regulation in PCM-containing 3DPC, while also highlighting that the thermal behavior of the printed material depends on the incorporation method, the mixture composition, and the test conditions.

Building on these studies, the present work investigated paraffin/sisal composite phase change fibers (PCFs) as a PCM carrier in 3DPC, with particular attention to rheological behavior and directional heat transfer. The PCFs were prepared by the vacuum impregnation method reported in our previous study [19]. Using these laboratory-prepared fibers as a raw material, the rheological properties of 3DPC incorporating PCFs were characterized, and specimens containing these fibers were fabricated. The heat-transfer behavior and microstructure of the resulting 3DPC were characterized and compared with those of sisal-fiber-reinforced 3DPC and fiber-free reference specimens. The pore structure and fiber distribution were analyzed to explore their possible relationships with the observed rheological and heat-transfer behaviors. The results provide experimental evidence for evaluating this material system under the tested conditions; its implications for building energy consumption require further evaluation at the component and building scales.

## 2. Materials and Methods

### 2.1. Materials

The phase change fibers used in this study were prepared using paraffin and sisal fibers (SFs) as raw materials. The paraffin was supplied by Shengbang Plastic Raw Material Factory (Dongying, China), with carbon (C) and hydrogen (H) as its primary elements. It has a density of 0.76 g/cm^3^ in its solid state and 0.86 g/cm^3^ in its liquid state, a melting point of 25 ± 2 °C, and a phase change enthalpy of at least 140 J/g. The sisal fibers were supplied by Guangxi Sisal Company (Nanning, China). The fibers had a length of 6 mm, a density of 1.18 g/cm^3^, and diameters of approximately 5–300 μm, with tensile strengths ranging from 470 to 720 MPa. The sisal fibers used in this study were the same as those used as the base material for the phase change fibers. Both the sisal fibers and the phase change fibers were stored in sealed containers at room temperature and were added to the mixtures in their air-dry state.

The phase change fibers were prepared using a vacuum impregnation (vacuum adsorption) method consisting of three steps [19]: (i) pretreatment, in which the sisal fibers were repeatedly rinsed with tap water to remove dust and dried at 60 °C for 24 h; (ii) vacuum impregnation, in which the dried fibers were immersed in molten paraffin (above its melting point of 25 ± 2 °C) and evacuated to −0.1 MPa for 1 h, during which the pressure difference drove the liquid paraffin into the fiber lumens with a paraffin-to-sisal mass ratio of 20%; and (iii) post-treatment, in which the impregnated fibers were spread flat on filter paper in a 60 °C oven, weighed after 1 h following cooling to room temperature and after each renewal of the filter paper, until a constant mass was reached, thereby removing paraffin that was not stably retained.

**Figure 1:**
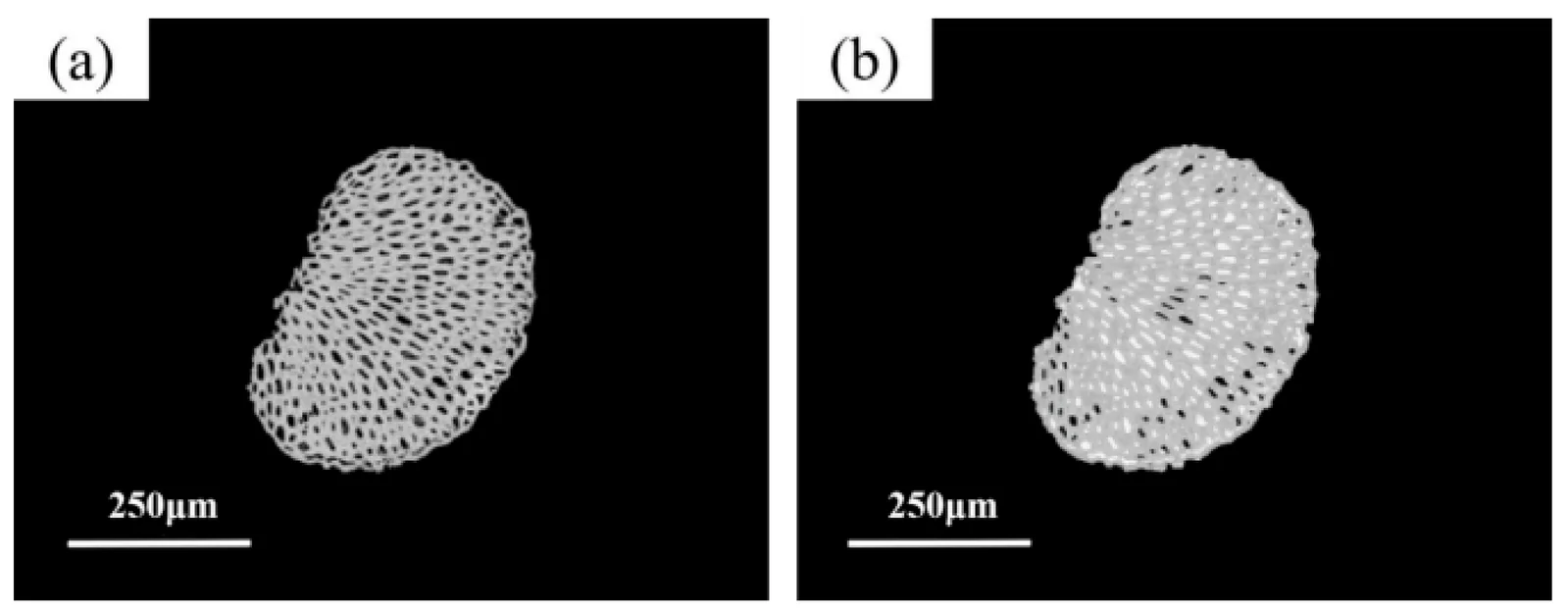
Nano-computed tomography (nano-CT) section images of (**a**) sisal fiber and (**b**) composite phase change fiber. Reproduced from Ref. [19] under the terms of the Creative Commons Attribution (CC BY) license.

**Figure 2:**
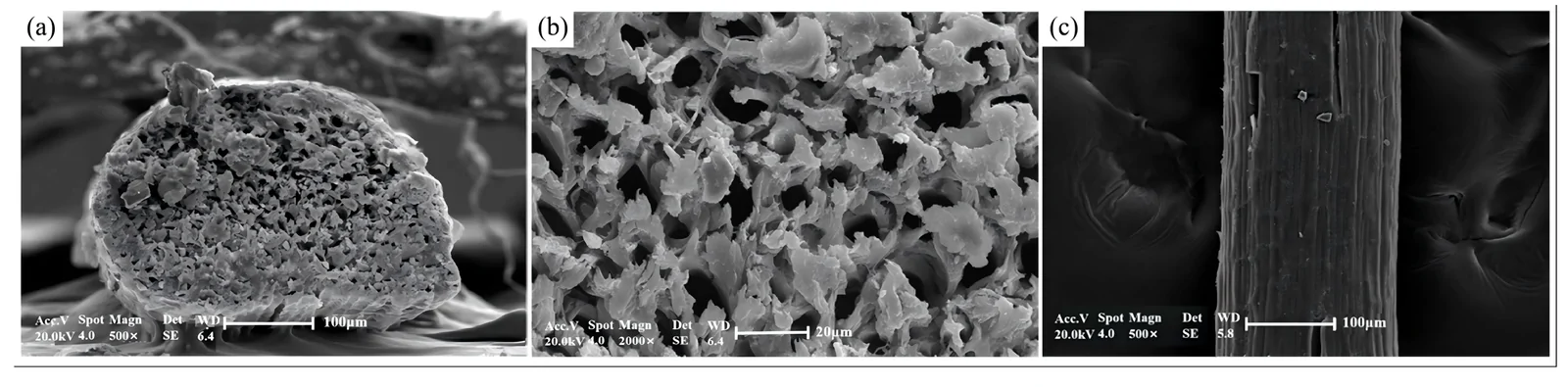
Scanning electron microscopy (SEM) images of sisal fibers: (**a**) cross-sectional view, (**b**) pores in the cross-section observed at high magnification and (**c**) longitudinal view. Reproduced from Ref. [19] under the terms of the Creative Commons Attribution (CC BY) license.

The resulting phase change fibers had a length of 6 mm and a density of 1.13 g/cm^3^, with paraffin accounting for approximately 20% of the sisal fiber mass. The water absorption rate of the resulting phase change fibers was 64.07% (by mass), as determined according to the procedure described in Ref. [35]. The paraffin is primarily distributed within the hollow conduits of the sisal fibers and on their surfaces, with about 90% of the stored paraffin residing within the hollow conduits [19]. The morphology of the sisal fibers and the resulting phase change fibers is shown in Figure 1 and Figure 2 [19].

Portland cement (Type PII 52.5R) was used in this study, supplied by Anhui Conch Cement Co., Ltd. (Wuhu, China). The cement had a density of 3.1 g/cm^3^ and a specific surface area of 0.363 m^2^/g determined using the Blaine air permeability method, and was verified to have acceptable stability. Silica fume used in this study was supplied by Yuanheng Water Purification Materials Factory (Gongyi, China). The silica fume has a density of 1.6 g/cm^3^ and a specific surface area of 10.557 m^2^/g, determined using the Brunauer–Emmett–Teller (BET) method. The chemical composition of the cement and silica fume is presented in Table 1. River sand supplied by Sobute New Materials Co., Ltd. (Nanjing, China), was used as the fine aggregate, with a maximum particle size of 1.18 mm.

A polycarboxylate-based high-performance superplasticizer (PCE) with a solid content of 30% was used. It was supplied by Jiangsu Sobute New Materials Co., Ltd. (Nanjing, China). Hydroxypropyl methylcellulose (HPMC), purchased from Suzhou Saimo New Material Technology Co., Ltd. (Suzhou, China), was used to adjust the cohesion of the paste. Its molecular formula is C_18_H_38_O_14_, with a density of 1.39 g/cm^3^ and a viscosity of 75,000 Pa·s.

### 2.2. Mix Design

#### 2.2.1. Mix Proportions and Paste Preparation for Rheological Performance Study

To investigate the effects of fiber type and dosage on the rheological properties of 3DPC, experiments were conducted using the mix proportions shown in Table 2.

For the mix proportions presented in Table 2, equal masses of sisal fibers and phase change fibers were used at each nominal dosage level. Because the two fibers differ in density, the corresponding fiber volume fractions differed by approximately 4%; this difference is acknowledged as a limitation of the present study. To account for the reduction in paste flowability associated with fiber water absorption, the mixing water was adjusted for the fiber-reinforced mixtures by considering the measured water absorption rates of the sisal fibers (93.24%) and phase change fibers (64.07%). Accordingly, at the same fiber dosage, less additional water was required for the phase change fiber mixtures because of their lower water absorption capacity.

The mixtures were prepared in a standard cement mortar mixer (JJ-5 type, Wuxi Jianyi Instrument & Machinery Co., Ltd., Wuxi, China) with a planetary mixing action, in accordance with GB/T 17671 [36]. The mixer had two speed settings: at the low-speed setting, the mixing blade rotated at 140 ± 5 r/min about its own axis and revolved at 62 ± 5 r/min about the mixer axis; at the high-speed setting, the corresponding values were 285 ± 10 r/min and 125 ± 10 r/min, respectively. The specimens for rheological testing of 3DPC were prepared as follows: (1) The dry materials (cement, silica fume, sand, HPMC, etc.) were mixed at low speed for 1 min to ensure uniform mixing of the dry ingredients. (2) The mixture of water and superplasticizer was added, and mixing was continued at low speed for 1 min. (3) Mixing was continued at low speed for another 1 min, during which the fibers, when required, were loosened by hand and gradually sprinkled into the running mixer. (4) The mixtures were then mixed at high speed for 1 min, followed by mixing at low speed for 30 s. The 3DPC specimens prepared according to the above procedure were used for rheological performance testing.

#### 2.2.2. Mix Proportions and Specimen Preparation for Thermal Performance Study

**Figure 3:**
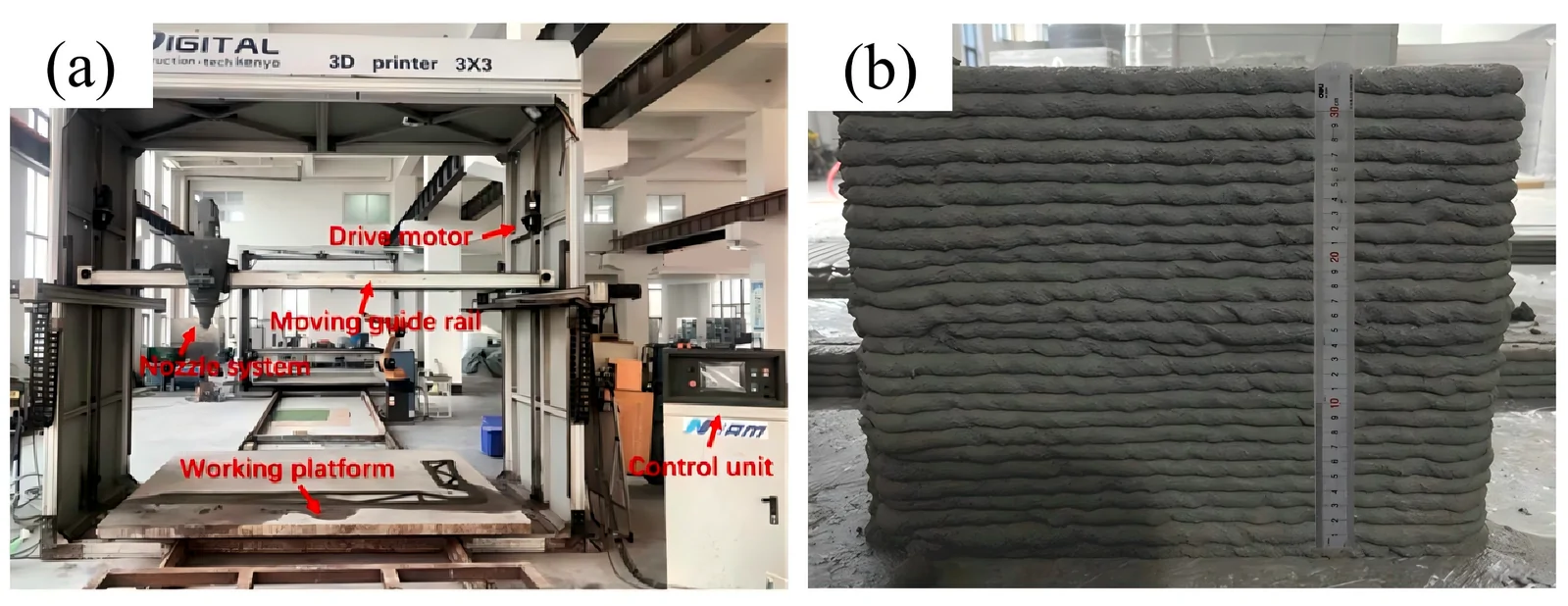
(**a**) Concrete 3D printer; (**b**) printed specimens.

Rheological screening of mixtures with varying fiber dosages revealed that both the static and dynamic yield stresses generally increased with increasing fiber dosage, whereas the plastic viscosity decreased. Mixtures with a nominal fiber dosage of 1.0% exhibited rheological characteristics compatible with the subsequent 3D printing process, providing an appropriate balance between resistance to deformation after extrusion and the flow behavior required for extrusion. Accordingly, F0.0%, SF1.0%, and PCF1.0% were selected from Table 2 for the subsequent printing and thermal performance tests. F0.0% served as the fiber-free reference, while SF1.0% and PCF1.0% were used to compare the effects of sisal fibers and phase change fibers at the same nominal fiber dosage. The printing was carried out using a 3 m × 3 m × 3 m gantry-style 3D printer produced by Nanjing Jiayi Precision Machinery Manufacturing Co., Ltd. (Nanjing, China) as shown in Figure 3a, with the printed specimens shown in Figure 3b. A circular print head with a diameter of 20 mm was used. The extrusion speed of the print head was 70 r/min, and the movement speed was 150 mm/s. The printing interval between adjacent layers was set to 13 s. The height of each printed layer was approximately 12 mm, and the width was about 24 mm.

**Figure 4:**
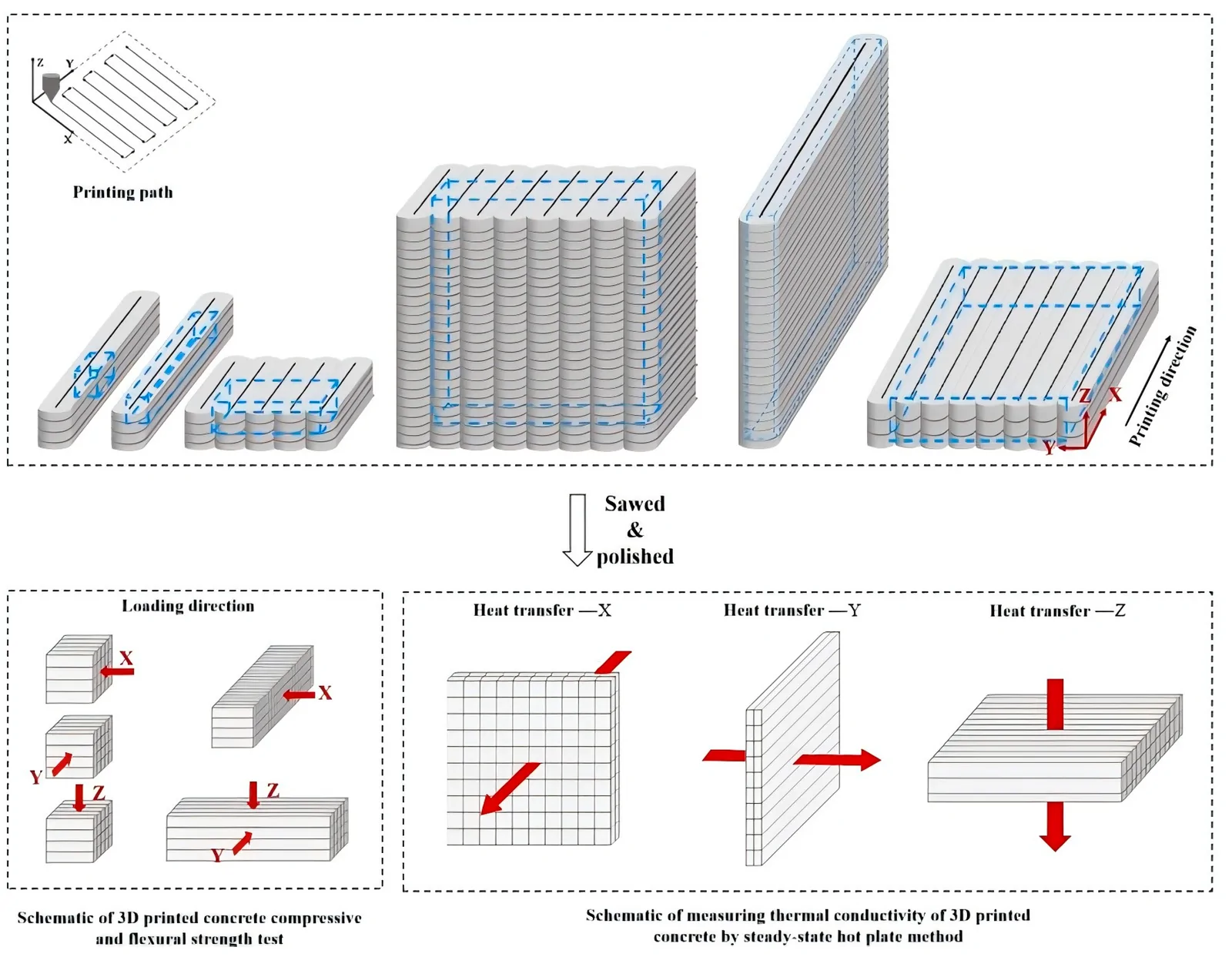
Schematic diagram of the cutting of the printed cement-based composite specimens.

By adjusting the dosage of admixtures, it was ensured that the fiber-reinforced 3DPC could be printed properly. After printing, the specimens were covered with plastic sheets and cured in an environment with a relative humidity of over 95% for 24 h. As illustrated in Figure 4, the specimens were cut with a concrete cutting machine by Jiangsu Yuemei Stone Co., Ltd. (Nanjing, China) to obtain test specimens for thermal conductivity (300 mm × 300 mm × 30 mm) and thermal insulation performance (40 mm × 40 mm × 15 mm). The same cutting layout and sampling orientations shown in Figure 4 were adopted for all 3DPC mixtures. After cutting, the printed specimens were polished to ensure that the dimensions met the requirements and that the surfaces were smooth. The test specimens were then transferred to a constant-temperature and humidity chamber (relative humidity of 90 ± 2%, temperature of 20 ± 2 °C) and cured until the designated testing age.

### 2.3. Testing Methods

#### 2.3.1. Dry Bulk Density Measurement

The dry bulk density of the printed and cast cement-based composite specimens was determined with reference to the method described in JG/T 266-2011, Foam Concrete [37]. The dry bulk density was calculated from the dry mass and measured external volume of each specimen.

#### 2.3.2. Rheological Performance Testing Method

The rheological performance of 3DPC was measured using the Brookfield RS-4 rheometer (Brookfield Engineering Laboratories, Inc., Middleboro, MA, USA). A four-bladed vane spindle (VT 20-10, vane diameter 20 mm, vane height 10 mm) was selected for the test. A constant shear rate of 0.1 s^−1^ was maintained for 3 min, and the peak shear stress was taken as the static yield stress of the cement-based composite material [38]. The static yield stress of 3DPC was also measured at 10 min, 20 min, 30 min, and 40 min after adding water using the same testing method [39].

The following procedure was used to measure the dynamic yield stress and plastic viscosity of 3DPC: over a period of 60 s, the rotor speed was linearly increased from 0 s^−1^ to 50 s^−1^; in the next 60 s, the rotor speed was linearly decreased from 50 s^−1^ to 0 s^−1^. The Bingham equation τ = τ_0_ + ηγ was used to fit the curve of decreasing shear rate, where τ is the shear stress, τ_0_ is the dynamic yield stress, η is the plastic viscosity, and γ is the shear rate [40].

#### 2.3.3. Thermal Performance Testing Method

The thermal conductivity of 3DPC was measured using the heat flow thermal conductivity meter (Netzsch-Gerätebau GmbH, Selb, Germany, model: HFM436/3/1E), following the GB/T 10294-2008 standard, ‘Determination of Steady-State Thermal Resistance and Related Properties of Thermal Insulation Materials—Guarded Hot Plate Method’ [41]. During testing, the specimen was placed between the hot plate and the cold plate, with each plate connected to a thermostatic water bath to maintain temperature stability (the cold plate temperature was kept constant at 25 °C, and the hot plate temperature was kept constant at 35 °C).

The thermal conductivity was calculated using Equation (1):

(1) $$k=\frac{I^{2}\mathrm{Rd}}{A_{h}∆t},$$

where *k* is the thermal conductivity of the specimen (W/(m·K)), *I* is the current through the heating element (A), *R* is its electrical resistance (Ω), *d* is the specimen thickness (m), *A_h_* is the effective heating area (m^2^), and ∆*t* is the temperature difference between the hot and cold plates (K).

Since specimen moisture content significantly affects thermal conductivity [42], all specimens were dried to constant mass in an air-circulating oven at 60 °C before thermal conductivity measurements. Previous studies have shown that this temperature does not cause the rapid evaporation of the bound water in 3DPC while markedly accelerating the drying process without causing surface cracking of the specimens [43].

Following the method proposed in reference [31], the anisotropic degree (AD) was used to characterize the effect of the printing process on the thermal conductivity of 3DPC and was calculated using Equation (2):

(2) $$\mathrm{AD}=\frac{{\mathrm{maximum}}_{k}-{\mathrm{minimum}}_{k}}{{\mathrm{minimum}}_{k}}\times 100\%,$$

In the equation, *maximum_k_* represents the maximum thermal conductivity of the printed specimen in W/(m·K), and *minimum_k_* represents the minimum thermal conductivity of the printed specimen in W/(m·K). A value of AD closer to 0 indicates a smaller anisotropy in the heat-transfer behavior of the printed specimen.

**Figure 5:**
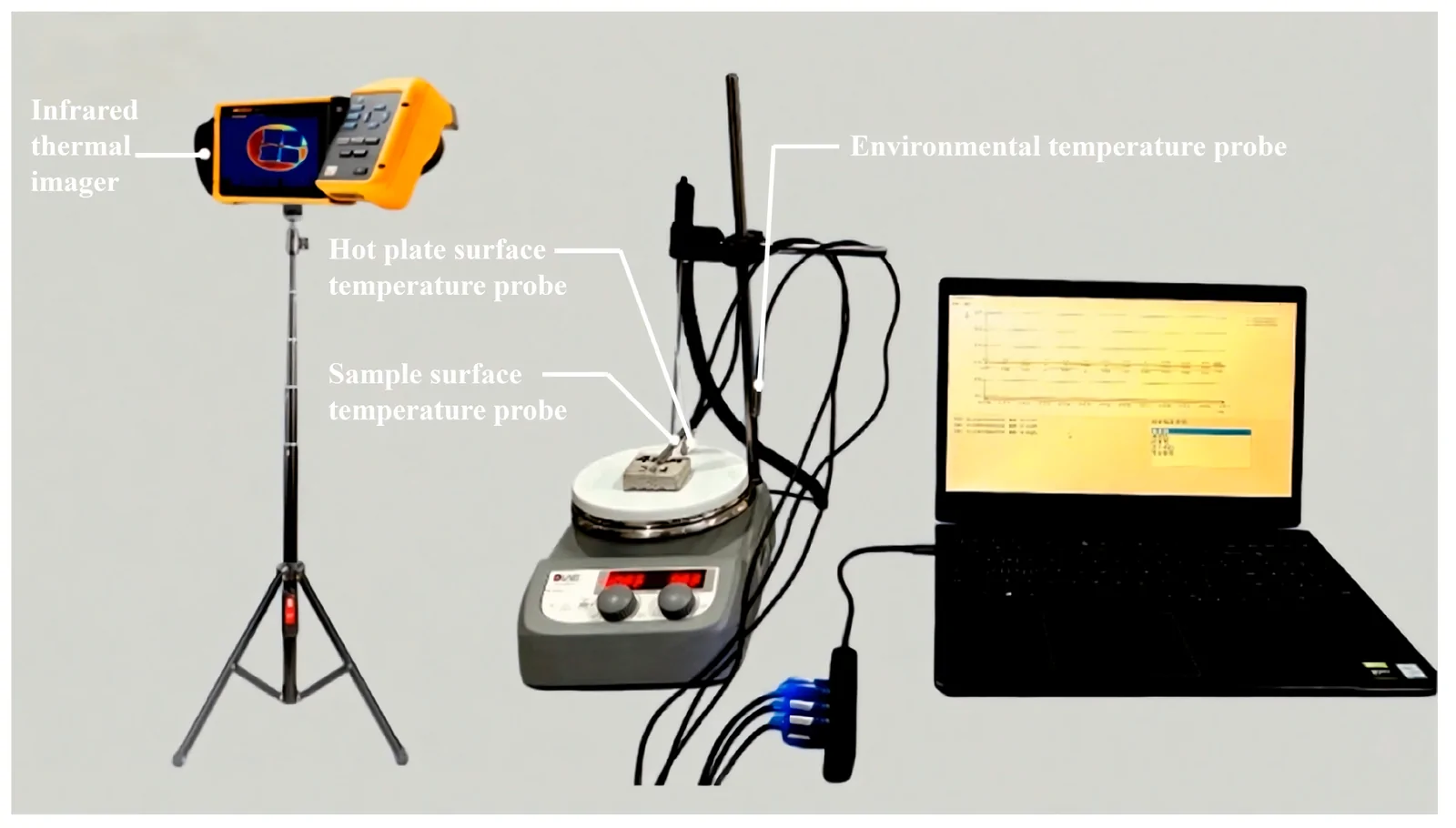
Experimental setup for testing thermal performance.

The thermal insulation performance of 3DPC was tested using the experimental setup shown in Figure 5. The thermal insulation performance was evaluated through a single heating and cooling cycle, with the heating and cooling times set to 20 min each. Given the experimental and time constraints, this evaluation was limited to a preliminary comparison of the temperature responses under the specified test conditions, and no independent replicate tests were performed. To collect real-time temperature variations during the heating and cooling process, three thermocouples were placed: one at the center of the hot plate surface to record the temperature of the hot plate; one 20 cm away from the hot plate to record the ambient temperature during the test; and another attached to the center of the surface of the test specimen to record the temperature of the specimen. The thermocouples recorded data every 0.1 s and transmitted it to a computer. Before testing, the specimens were dried to a constant weight at 60 °C.

The thermal insulation anisotropy index (I_h_) was used to characterize the effect of the printing process on the thermal insulation performance of 3DPC, which can be calculated using Equation (3):

(3) $$I_{h}=\frac{{\mathrm{maximum}}_{T}-{\mathrm{minimum}}_{T}}{{\mathrm{minimum}}_{T}},$$

In the equation, *maximum_T_* represents the maximum rate of temperature change in the printed specimen during the heating process, in °C/min. *minimum_T_* represents the minimum rate of temperature change in the printed specimen during the heating process, in °C/min. The closer the value of *I_h_* is to 0, the smaller the difference in heat insulation anisotropy of the printed specimen.

#### 2.3.4. Pore Structure Testing Method

The porosity and pore size distribution of 3DPC specimens were measured using an automatic mercury intrusion porosimeter (MIP; Micromeritics Instrument Corporation, Norcross, GA, USA, model: AutoPore IV 9520). The specimens were cured in a standard curing chamber at 20 ± 2 °C and 90% relative humidity for 28 days. Specimens were then cut from the printed filaments, avoiding the printed interfaces wherever possible, and oven-dried at 60 °C to constant mass to remove free water from the pores.

#### 2.3.5. Fiber Distribution Testing Method

The actual dispersion of fibers in 3DPC was observed using industrial X-ray computed tomography (CT) (Y.CT PRECISION S-type; YXLON International GmbH, Hamburg, Germany). The specimen size scanned was 40 × 40 × 40 mm^3^, and 800 slice images were captured for each specimen with a resolution of 1024 × 1024 pixels. The spatial distribution of the fibers in the specimen was analyzed using Dragonfly software (version 2024.1) after three-dimensional reconstruction.

**Figure 6:**
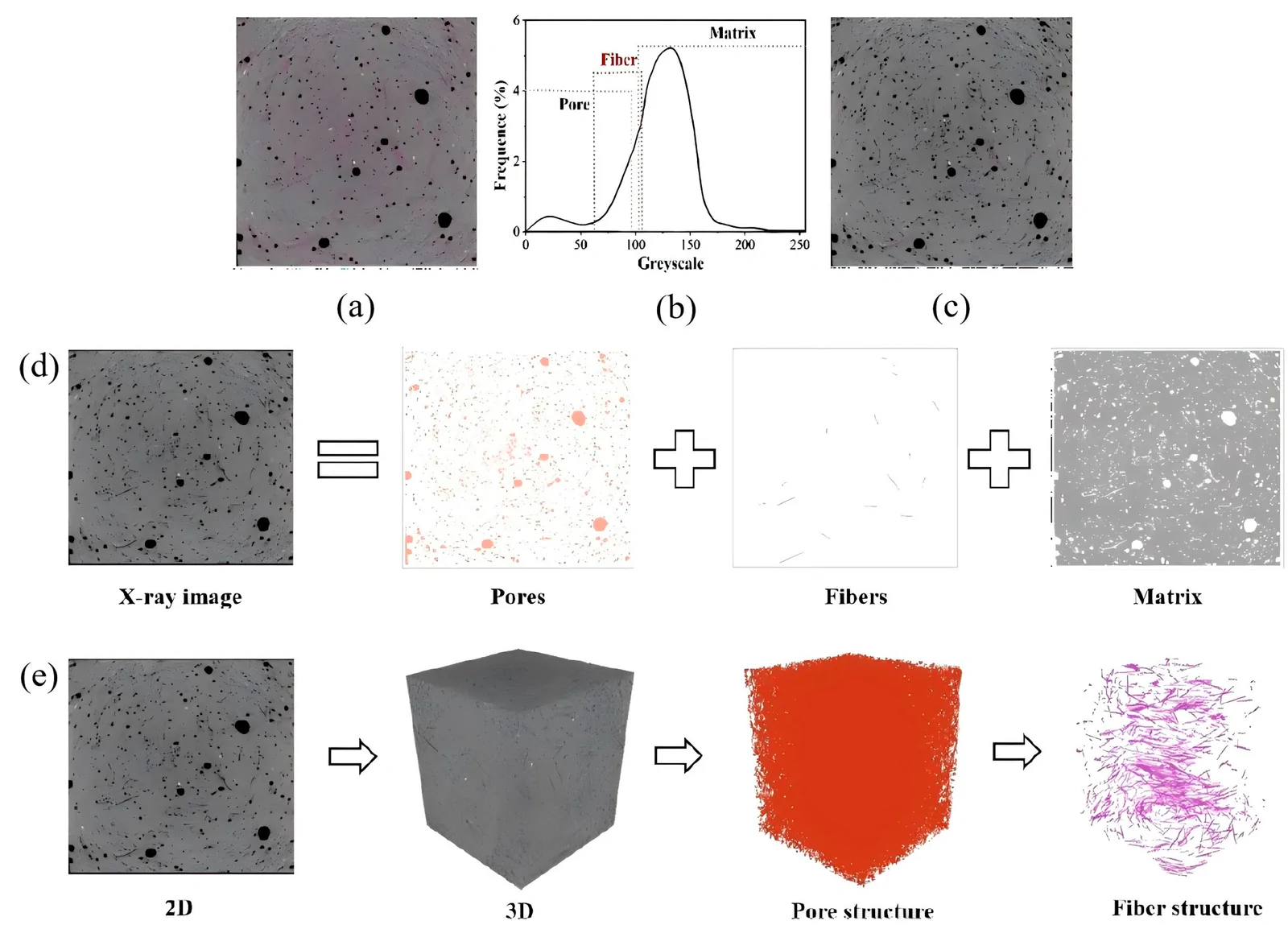
X-ray image processing: (**a**) threshold segmentation method; (**b**) greyscale of fibers overlapping partially with that of pores; (**c**) deep learning result; (**d**) the diagram of separating the X-ray image into pores, fibers and matrix; (**e**) 2D and 3D X-ray scanning of concrete.

Since the grayscale of SFs and PCFs was similar to that of the pores, it was difficult to distinguish fibers and pore regions in the image using conventional grayscale thresholding methods, as shown in Figure 6a,b. The commonly used section fluorescence labeling method [44,45] was also ineffective in reflecting the three-dimensional spatial distribution of fibers in the paste. Therefore, this study adopted deep learning methods to analyze the spatial distribution of fibers. Deep learning is a visual classification method involving convolutional neural networks (CNN) technology [46]. Using grayscale grading and fiber phase patterns, fibers, paste, and pores were manually distinguished in five images. The differentiated images were then input into a segmentation mapping training network, and the fiber, paste, and pore differentiation effects in the remaining slice images were observed. This process was repeated until the output matched the expected target, as shown in Figure 6c, with fibers represented in pink. A specimen containing 1.5 vol% PCFs was used as the standard specimen, as shown in Figure 6d,e. The trained deep learning model was then applied to CT image processing of all specimens.

**Figure 7:**
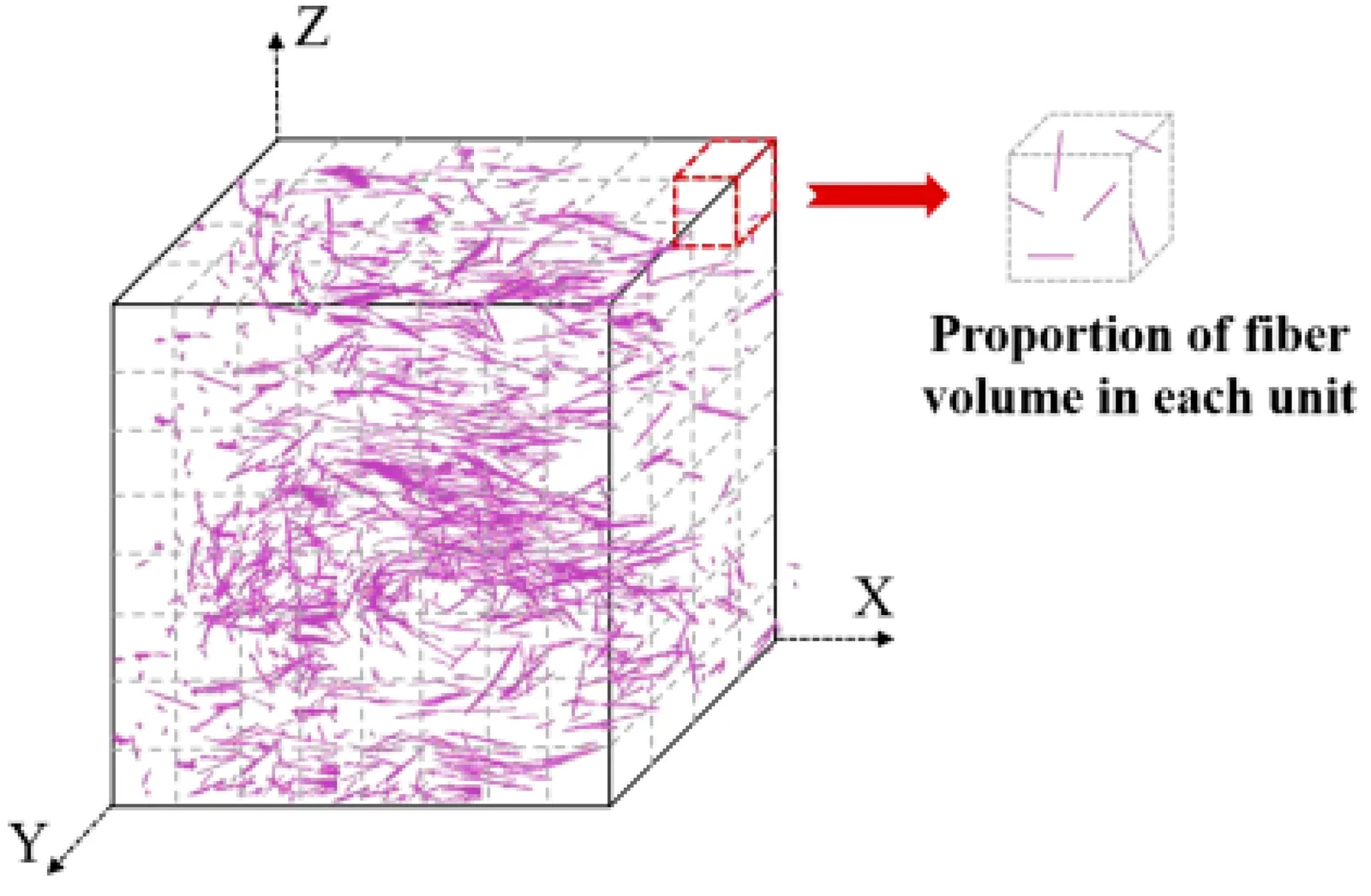
Illustration of fiber dispersion.

As shown in Figure 7, the three-dimensional reconstructed images of the fibers were sliced into 512 units, each measuring 5 × 5 × 5 mm^3^, and the fiber volume fraction within each unit was calculated.

## 3. Results and Discussion

### 3.1. Rheological Performance Test Results

#### 3.1.1. Static Yield Stress

**Figure 8:**
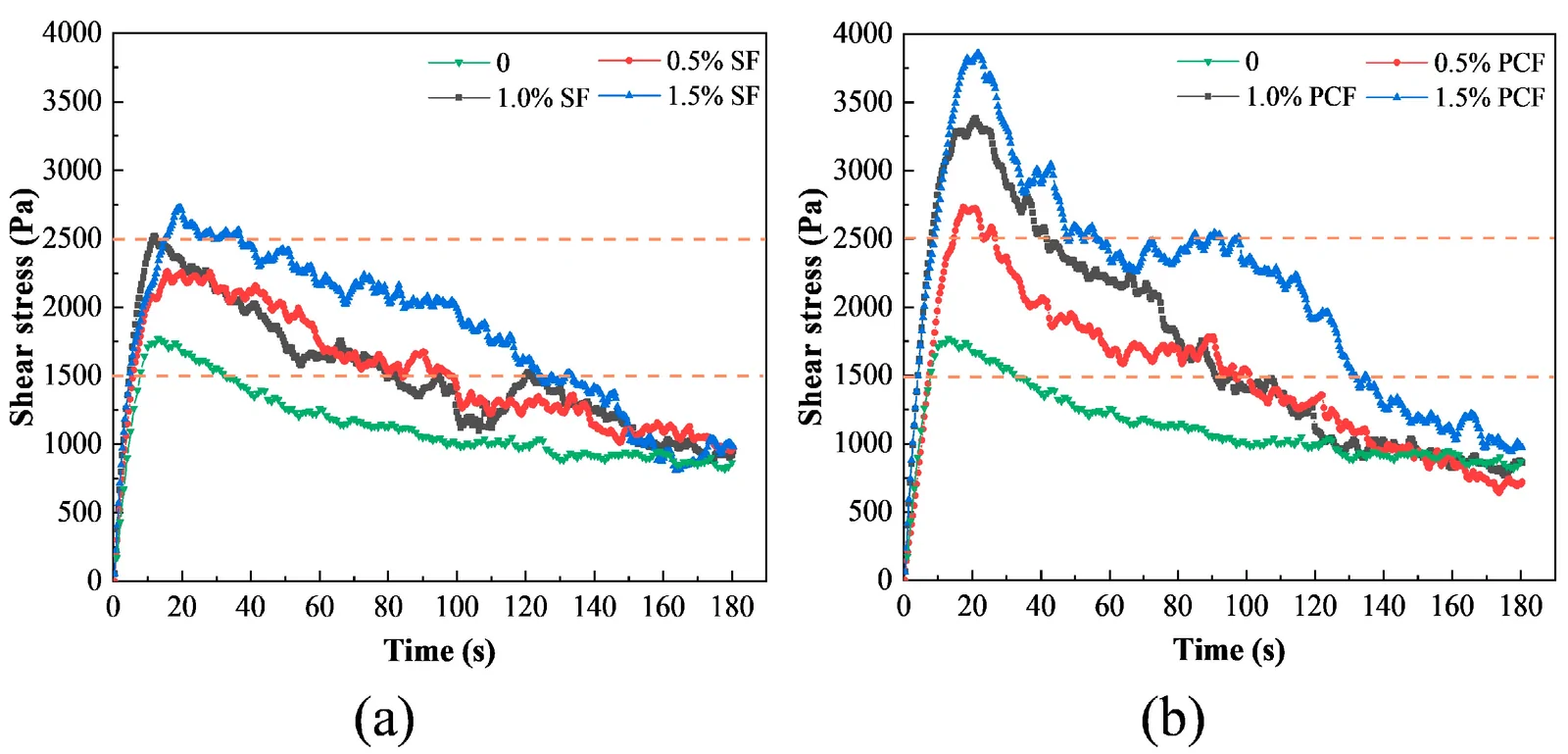
Static yield stress: (**a**) shear stress curves of mortar containing sisal fiber; (**b**) shear stress curves of mortar containing composite phase change fiber.

Based on the mix proportions in Table 2, the relationship between shear stress and fiber content in 3DPC was examined at 10 min after water addition, and the results are shown in Figure 8a,b. From Figure 8a, it can be seen that under the same time conditions, as the sisal fiber content increases, the shear stress of 3DPC also increases correspondingly. At the same fiber dosage, as the elapsed shearing time increases, the shear stress reaches a peak and then gradually decreases. This response is consistent with the yielding behavior of a yield-stress fluid, with the peak stress corresponding to the static yield stress. The subsequent decrease in shear stress is associated with the progressive breakdown of the internal structure under continued shearing [47]. From Figure 8b, it can be observed that phase change fibers exhibit the same pattern of behavior as sisal fibers.

**Figure 9:**
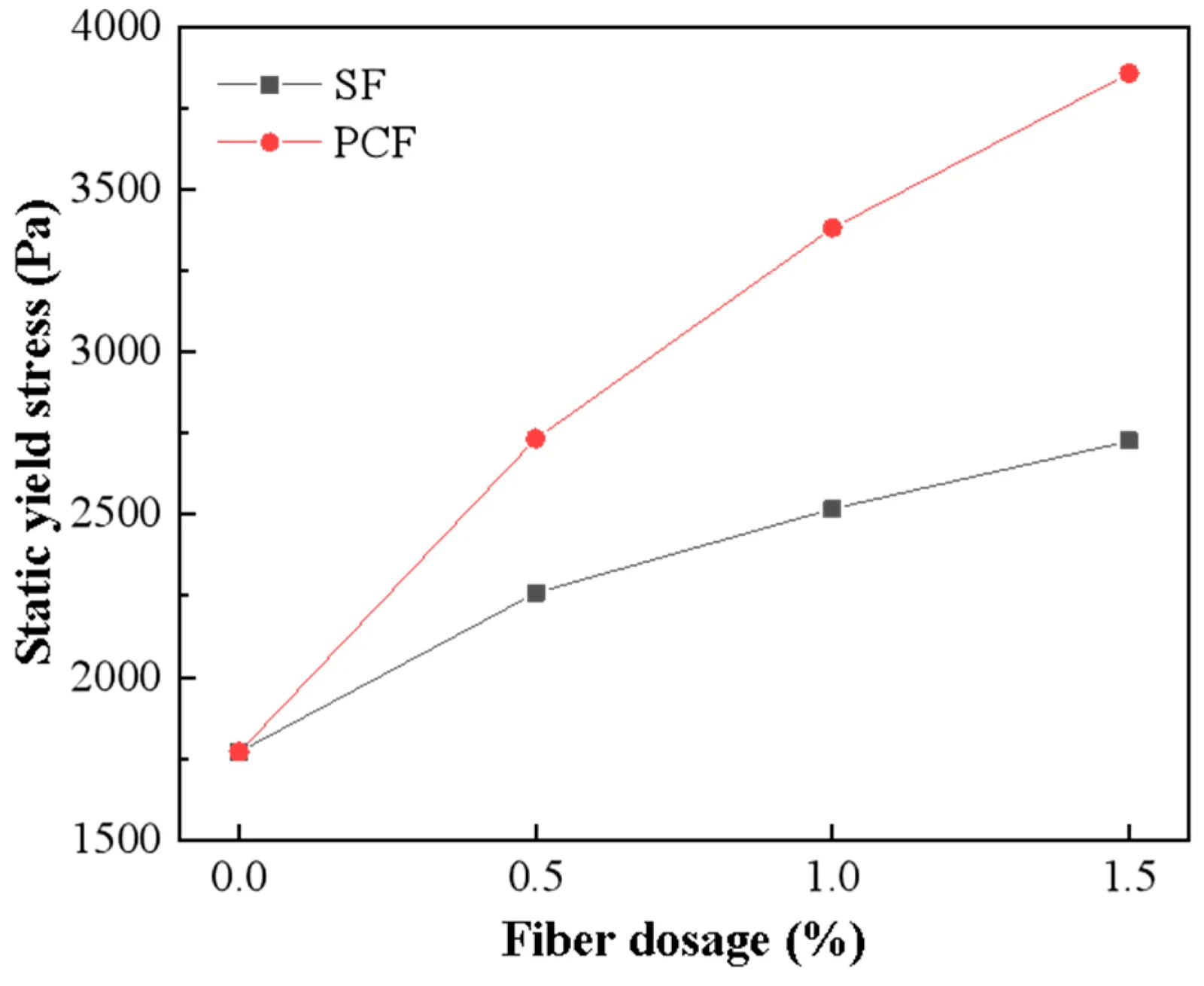
Static yield stress versus fiber dosage.

In this study, the static yield stress measured at 10 min after water addition is referred to as the initial static yield stress. The effects of different fiber types and dosages on the initial static yield stress were compared, and the results are shown in Figure 9. From Figure 9, compared to fiber-free 3DPC, the initial static yield stress of fiber-reinforced 3DPC significantly increases. After the introduction of fibers into the 3DPC paste, even with the addition of extra water, the fiber surface effects and the interlocking interactions still have a certain influence on the rheological performance of 3DPC [48,49,50].

At the same nominal fiber dosage, the initial static yield stress of 3DPC containing phase change fibers is much higher than that of 3DPC containing sisal fibers, resulting in reduced workability and extrudability of 3DPC containing phase change fibers. This phenomenon is hypothesized to be due to some of the paraffin in the phase change fibers adsorbing onto the surface of the sisal fibers and thus improving the dispersion of the phase change fibers. To verify this, subsequent studies employed X-ray tomography to investigate the dispersion of fibers in 3DPC.

**Figure 10:**
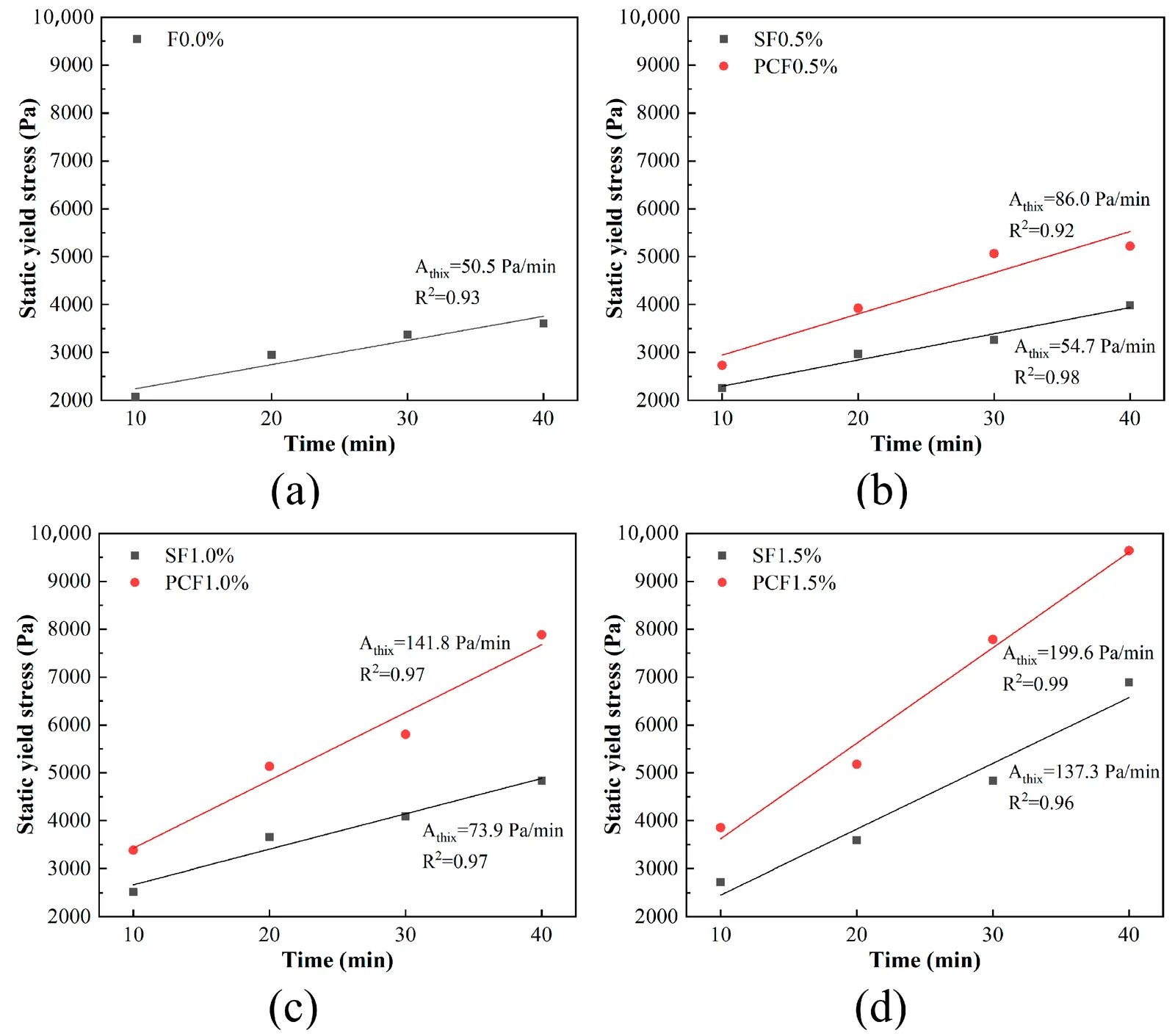
Evolution of static yield stress of cement-based composites with time: (**a**) control group; (**b**) 0.5% fiber content; (**c**) 1.0% fiber content; (**d**) 1.5% fiber content.

To investigate the shape-retention ability of 3DPC, static yield stress was measured at 10, 20, 30, and 40 min after water addition, and the results are plotted in Figure 10. The test results in Figure 10 were fitted using the formula proposed by Roussel [47], τ_0_(t) = τ_0_ + A_thix_ × t, where τ_0_(t) is the static yield stress at time t, τ_0_ is the initial static yield stress, and A_thix_ represents the structuration rate induced by thixotropy (the rate of increase in static yield stress). It can be observed that, for the fiber dosages studied (0%, 0.5%, 1.0%, 1.5%), the static yield stress increases linearly over time, and the thixotropy is higher with an increase in fiber content. Both fiber types exhibit similar patterns in the evolution of static yield stress over time.

#### 3.1.2. Dynamic Yield Stress

**Figure 11:**
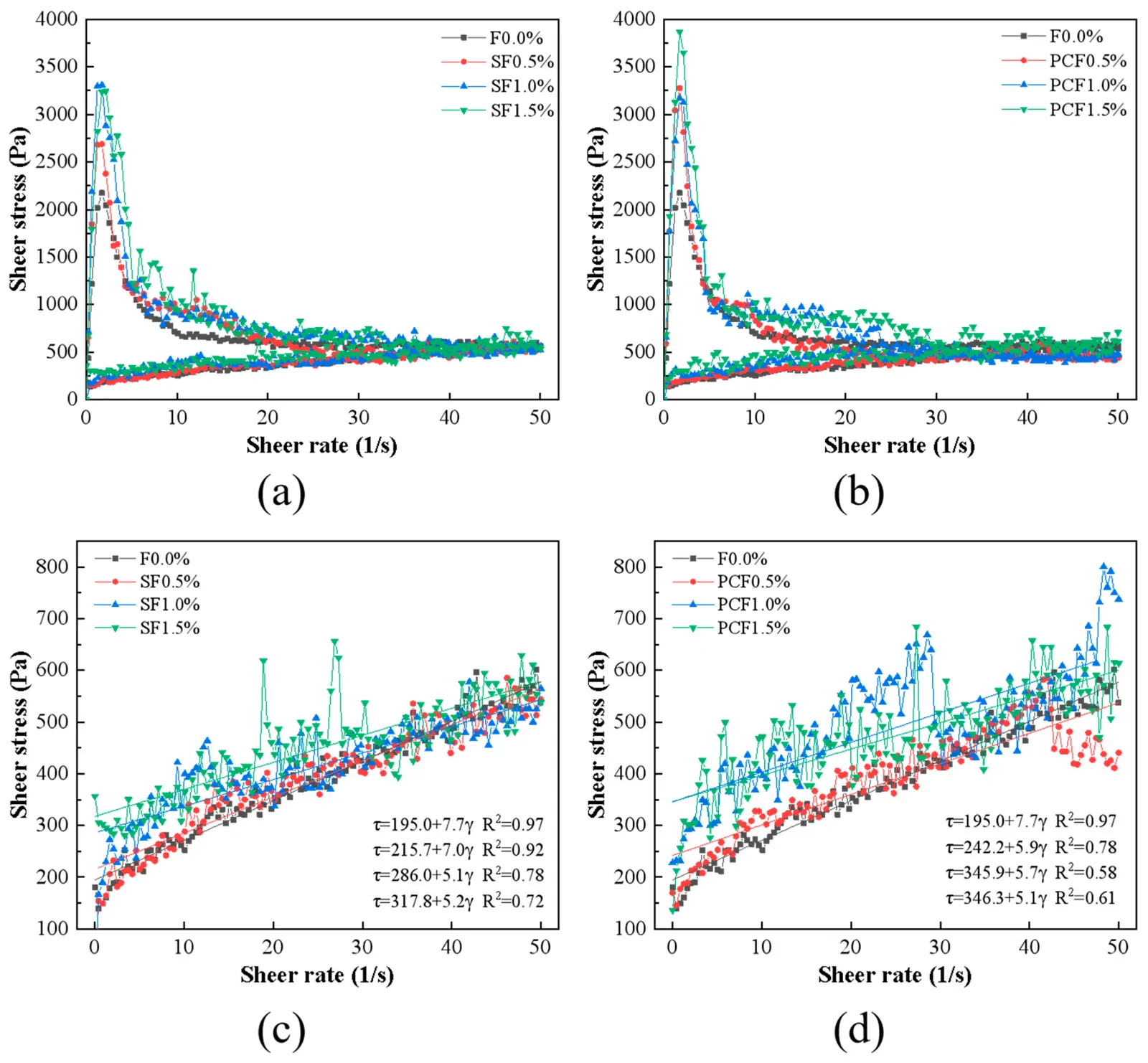
Dynamic yield stress: (**a**) effect of sisal fiber dosage; (**b**) effect of phase change fiber dosage; (**c**,**d**) fitted Bingham model.

Dynamic yield stress primarily characterizes the extrudability of 3DPC paste [51]. The dynamic yield stress of 3DPC containing sisal fibers and phase change fibers, measured 10 min after water addition, is shown in Figure 11a,b. The Bingham model was used to fit the descending segment of the test curves in Figure 11a,b, resulting in Figure 11c,d.

**Figure 12:**
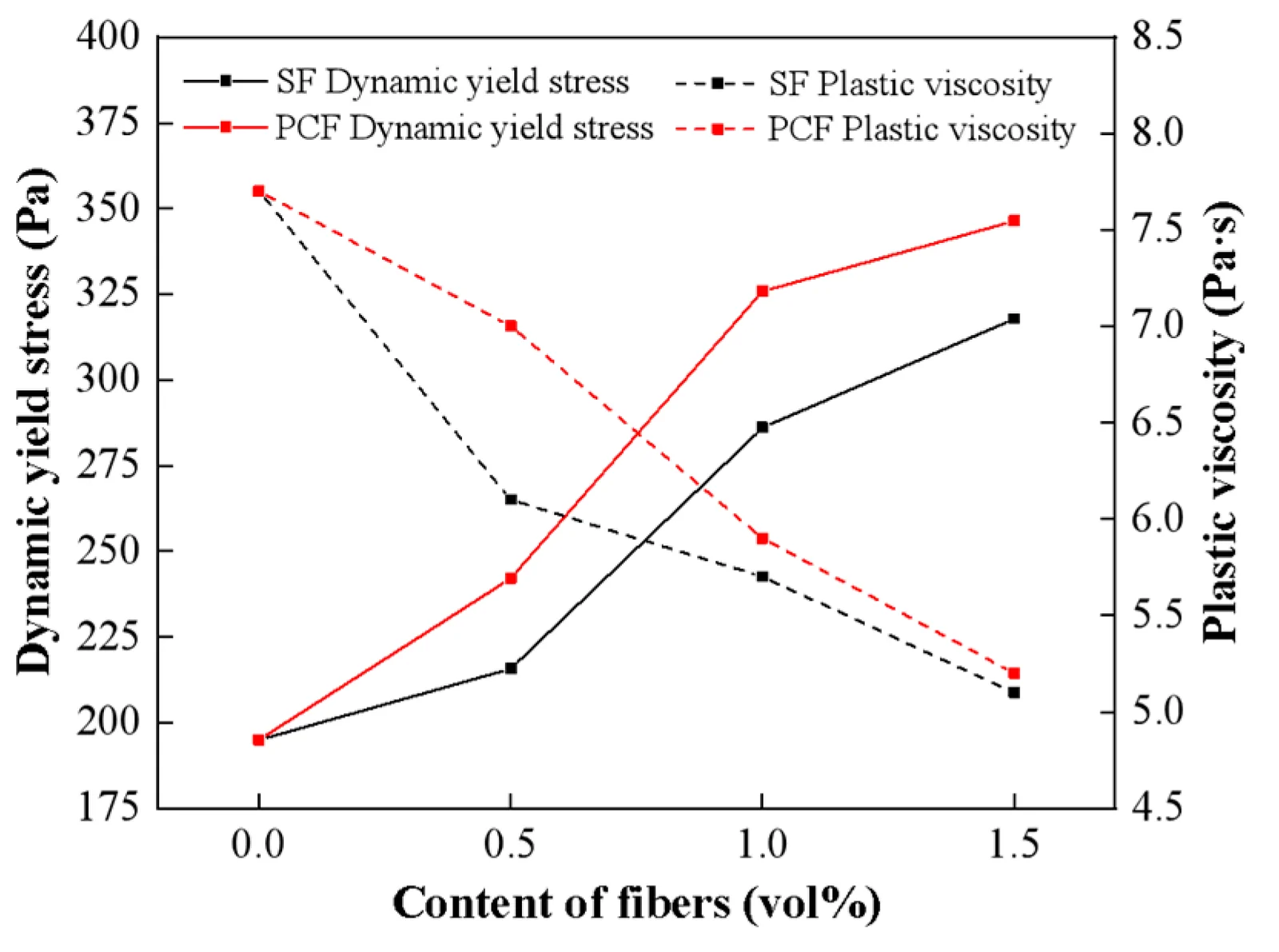
Dynamic yield stress and plastic viscosity as a function of fiber dosage.

Based on the fitting results in Figure 11, the effects of fiber type and dosage on the plastic viscosity and dynamic yield stress of 3DPC are shown in Figure 12. From Figure 12, it can be seen that as the sisal fiber content increases, the dynamic yield stress increases from 200 Pa to 320 Pa, similar to the trend observed for static yield stress. Similar trends have been observed in other studies [16]. The plastic viscosity decreases from 7.7 Pa·s to 5.1 Pa·s, which is likely associated with shear-induced fiber reorientation during rheological testing. Under increasing shear, the fibers tend to align preferentially with the flow direction, thereby reducing fiber–fiber interlocking and resistance to relative motion within the paste [52,53]. Such alignment facilitates material flow and consequently lowers the apparent plastic viscosity. This interpretation is also consistent with the preferential fiber orientation observed in the printed specimens, as discussed in Section 3.3.3. Compared to 3DPC containing sisal fibers, the dynamic yield stress and plastic viscosity of 3DPC containing phase change fibers exhibit a similar trend with changes in fiber content.

### 3.2. Thermal Performance

**Figure 13:**
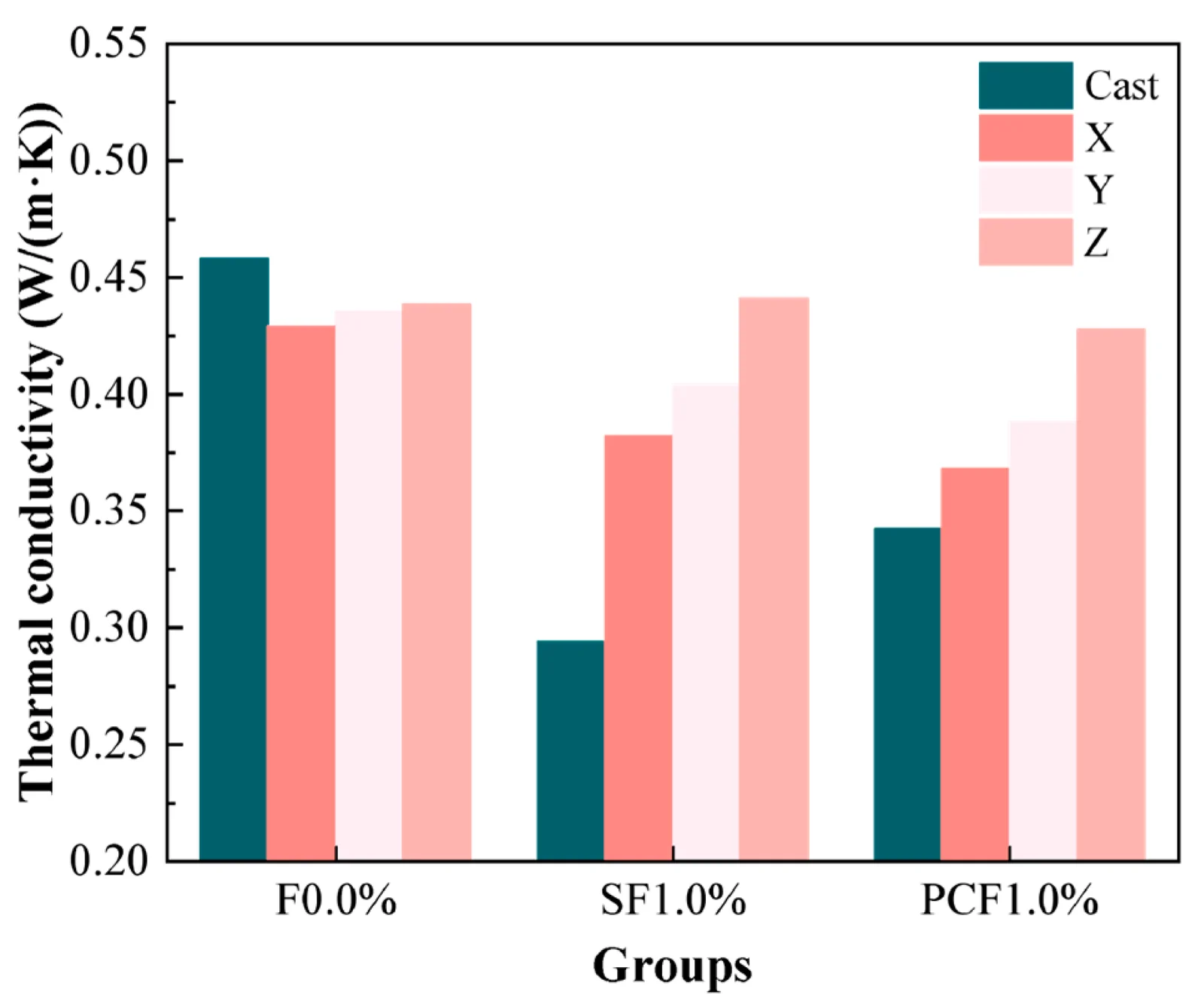
Thermal conductivity of printed and cast specimens of cement-based composite materials. X, Y, and Z denote the three heat-transfer directions of the 3D-printed specimens.

In this study, 3DPC was prepared and processed according to the mix proportions of selected groups shown in Table 2. To reflect the impact of the printing process, specimens with the same mix proportions were also cast. The thermal conductivity of both printed and cast specimens was tested, and the results are shown in Figure 13.

From Figure 13, it can be observed that the test results for the F0.0% group indicate that the thermal conductivity of the printed specimens is lower than that of the cast specimens, which is primarily related to the introduction of more pores during the printing process [54]. In the fiber-reinforced groups, the thermal conductivity of the printed specimens in the X, Y, and Z directions is higher than that of the cast specimens. This suggests that the printing process has an adverse effect on thermal conductivity, and a deeper analysis of the pore structure of fiber-reinforced 3DPC is required. When comparing fiber-reinforced groups with non-fiber groups, it can be found that, along the same heat-transfer direction, fiber-reinforced 3DPC exhibits a lower thermal conductivity. This is mainly because the thermal conductivity of both sisal fibers and the paraffin in phase change fibers is lower than that of the cement matrix, which, according to the principles of composite materials [55], leads to a decrease in the thermal conductivity of fiber-reinforced 3DPC. Moreover, the thermal conductivity of printed 3DPC specimens containing sisal fibers is higher than that of 3DPC specimens containing phase change fibers. This can be explained by the impact of fiber dispersion on the pore structure of 3DPC. The measured thermal conductivity values were substituted into Equation (2) to calculate the anisotropy degree (AD). The AD values for the F0.0%, SF1.0%, and PCF1.0% groups were 2.2%, 15.5%, and 16.2%, respectively. The incorporation of fibers increases the AD of 3DPC, which may be related to the directional distribution of the fibers.

**Figure 14:**
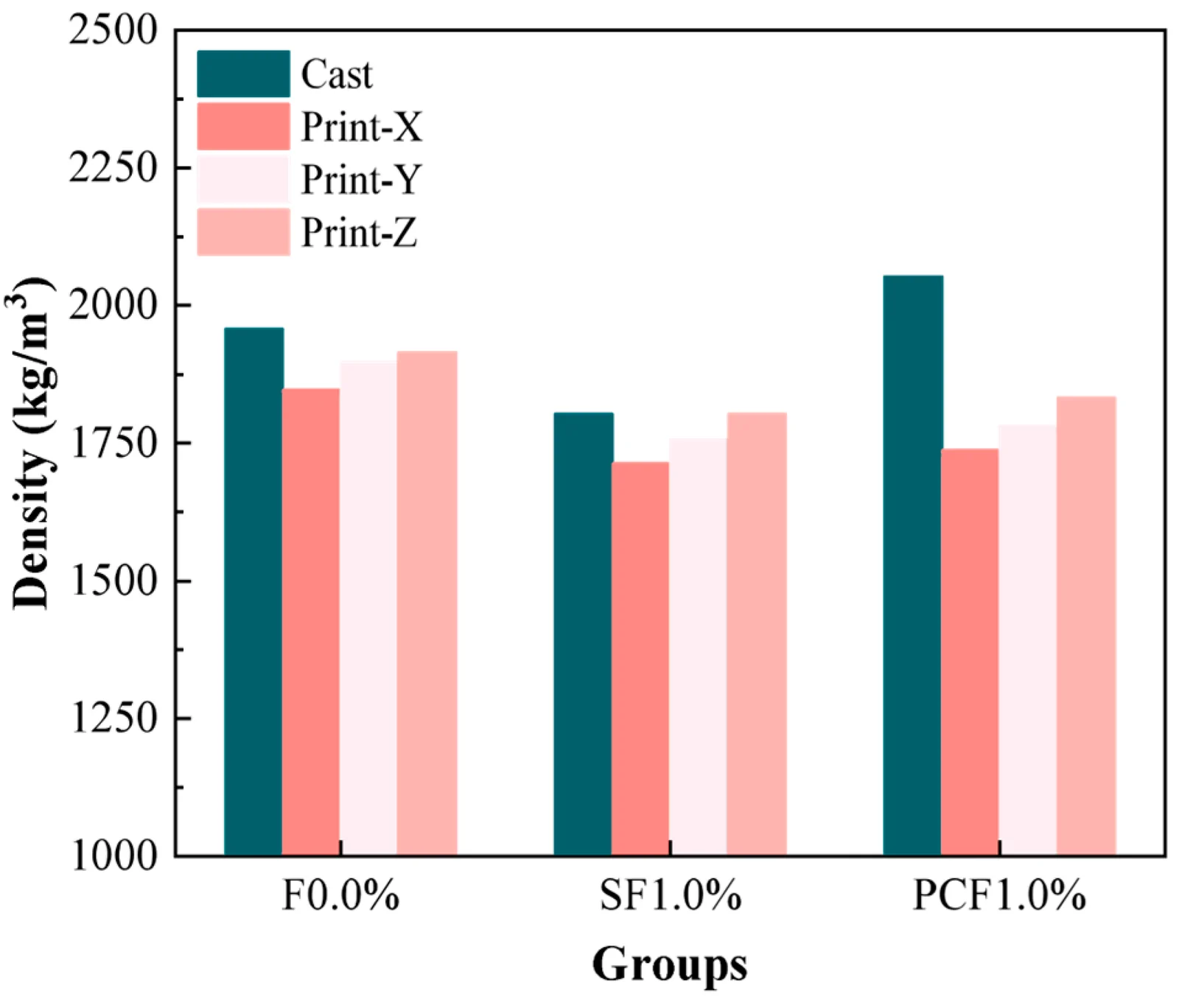
Density of printed and cast specimens of cement-based composite materials.

The dry bulk densities of the printed and cast cement-based composites are presented in Figure 14. In general, the printed specimens had lower dry bulk densities than their cast counterparts. For each mixture, the dry bulk densities of the printed specimen sets corresponding to the X, Y, and Z heat-transfer directions decreased in the order Z > Y > X. For a given sampling orientation, the SF1.0% specimens had the lowest dry bulk density, whereas the F0.0% specimens had the highest.

**Figure 15:**
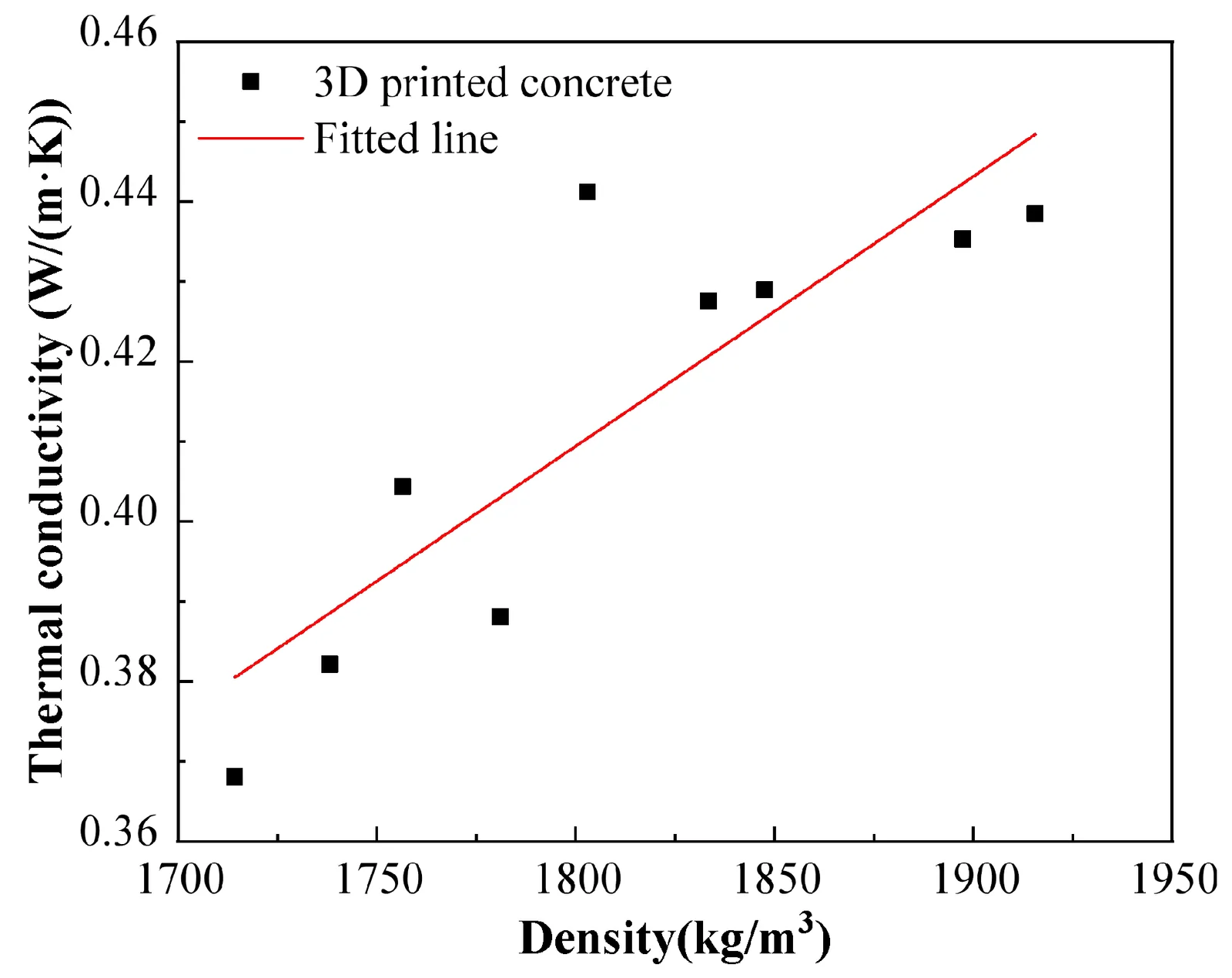
The relationship between thermal conductivity and density of cement-based composite printed specimens.

Figure 15 shows the relationship between thermal conductivity and dry bulk density for the printed specimens. Across the printed specimens investigated, thermal conductivity showed an overall positive correlation with dry bulk density. A linear regression of these data yielded the following:

*k*_3*D*_ = 0.000338ρ − 0.198, R^2^ = 0.73 (4)

where *k*_3*D*_ is the thermal conductivity of the printed specimens (W/(m·K)) and *ρ* is their dry bulk density (kg/m^3^).

Although this empirical relationship describes the overall trend among the investigated printed specimens, it cannot account for the higher thermal conductivity of the fiber-reinforced printed specimens relative to their cast counterparts, despite the generally lower dry bulk density of the former. Therefore, dry bulk density alone is insufficient to explain the reversed trend between the printed and cast specimens, and other structural factors may also contribute.

**Figure 16:**
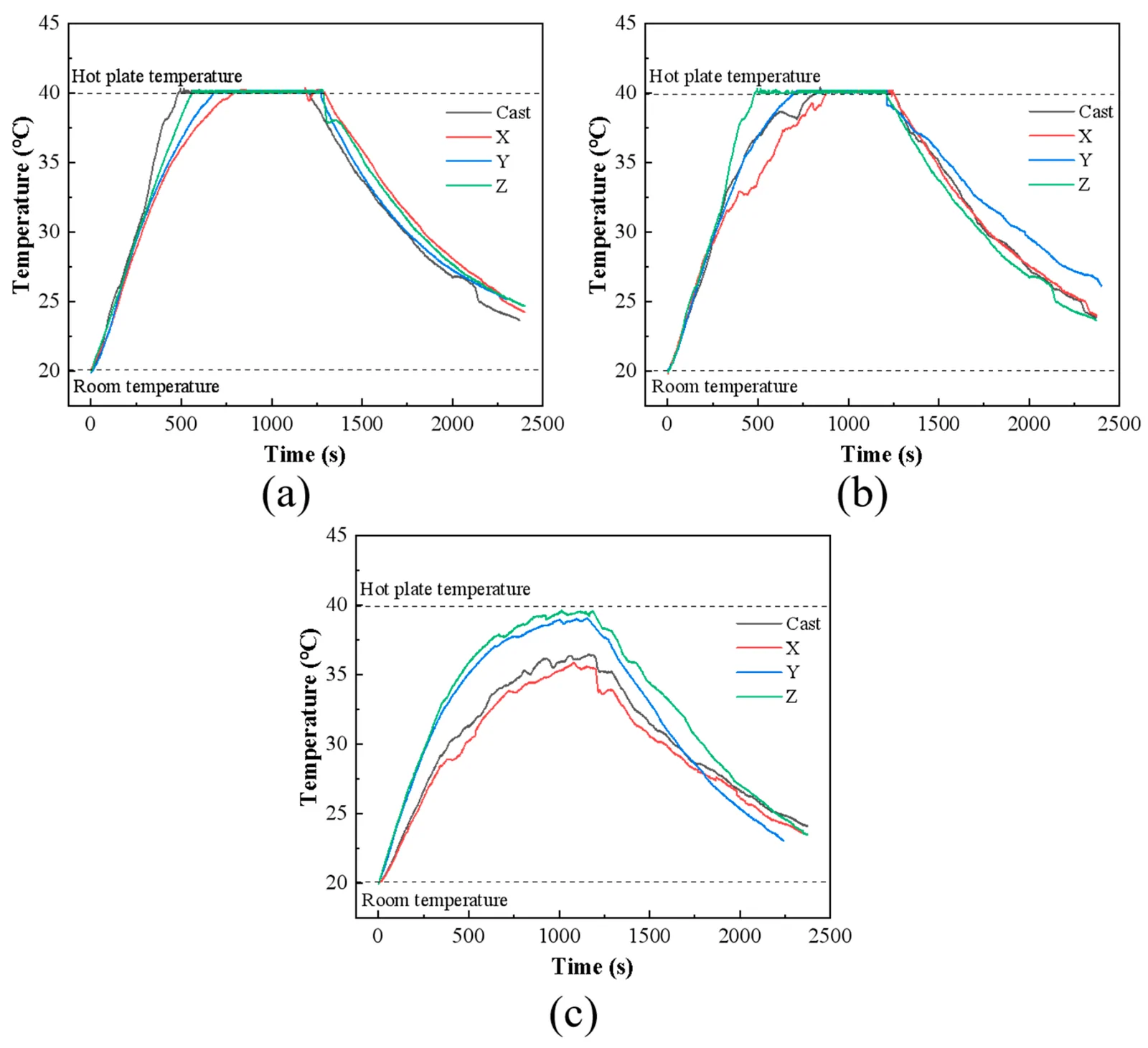
Surface temperature of printed and cast specimens of cement-based composite materials during the heating and cooling stages: (**a**) F0.0%; (**b**) SF1.0%; (**c**) PCF1.0%.

To compare the differences in thermal insulation performance between the three groups of printed and cast 3DPC specimens, thermocouples were used to measure the surface temperature variations in the specimens during the heating and cooling processes, and the results were plotted in Figure 16.

As shown in Figure 16a,b, within the specified heating time, the surface temperature of both the printed and cast specimens in the F0.0% and SF1.0% groups reached 40 °C. From Figure 16c, it can be observed that, within the specified heating time, the surface temperature of both the printed and cast specimens in the PCF1.0% group did not reach 40 °C. The highest surface temperatures of the cast specimen and printed specimens along the X, Y, and Z heat-transfer directions were 39.5 °C, 35.6 °C, 36.4 °C, and 38.8 °C, respectively, which were 0.5 °C, 4.4 °C, 3.6 °C, and 1.2 °C lower than 40 °C. These results indicate a lower surface temperature rise in the PCF1.0% specimens under the present heating conditions. The melting point of the paraffin (25 ± 2 °C), as reported in Section 2.1, lies within the temperature range covered by the test, suggesting that latent heat absorption during melting may contribute to this behavior.

The rate of temperature increase differs in various directions. When comparing the temperature change rates in different directions for the three groups of printed specimens, it was found that the heat-transfer rate along the Z direction was the fastest, followed by the Y direction, with the X direction being the slowest. The measured temperature change rates were substituted into Equation (3) to calculate the thermal insulation anisotropy index (I_h_). The I_h_ values for the F0.0%, SF1.0%, and PCF1.0% groups were 42.0%, 27.5%, and 23.9%, respectively.

### 3.3. Mechanism Analysis

The test results indicate that the rheological and thermal properties of 3DPC are closely related to its microstructure, necessitating an in-depth analysis of the microstructural characteristics of 3DPC.

#### 3.3.1. Pore Size Distribution

**Figure 17:**
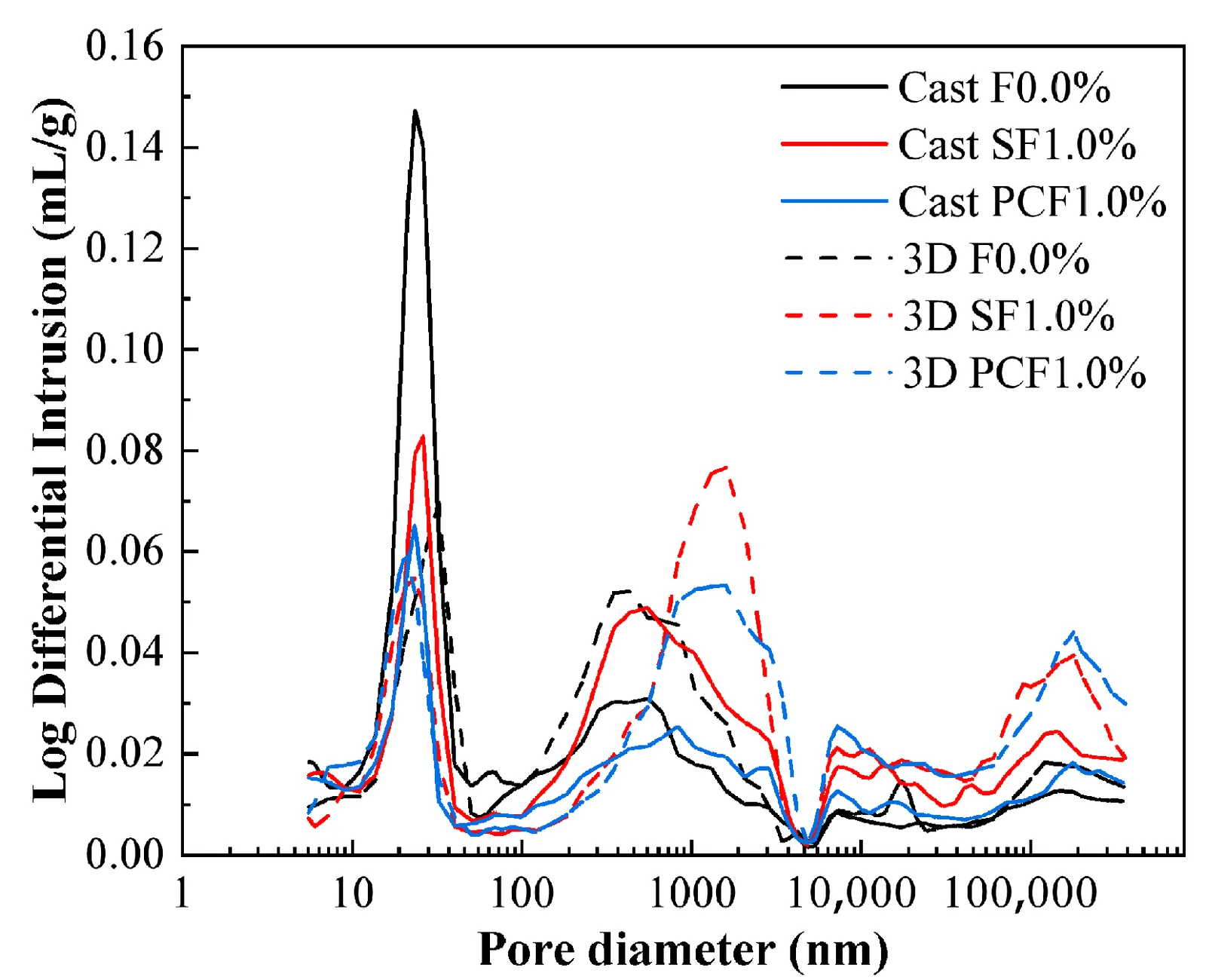
Pore distribution of printed and cast specimens of cement-based composite materials.

The results of the rheological and thermal performance tests suggest that the introduction of fibers influences the pore structure of the matrix due to the surface properties of the fibers and their interlocking effect. The pore size distribution and volume fraction of three types of 3DPC-printed and cast specimens were tested, and the results are shown in Figure 17.

From Figure 17, it can be observed that the pore size distribution curves of the three groups of 3DPC-printed and cast specimens exhibit three primary peaks. However, the peak positions and intensities vary among different groups. Based on the definition of pores in cement-based composites [56], pores can be classified into three categories: pores larger than 10,000 nm are considered voids/microcracks, those ranging from 50 to 10,000 nm are capillary pores/macropores, and those smaller than 50 nm are gel pores/mesopores. Generally, gel pores do not have an adverse effect on mechanical properties, but their small pore size prevents them from contributing significantly to thermal insulation. Capillary pores have some negative impact on mechanical performance but enhance thermal insulation. In contrast, voids negatively affect both mechanical and thermal properties.

For the F0.0% group, the pore size distribution curve of the cast specimen exhibits three primary peaks at 23.4 nm, 432.7 nm, and 144,343.7 nm, whereas the printed specimen exhibits peaks at 32.4 nm, 433.1 nm, and 120,700.9 nm. Compared to the cast specimens, the printed specimens exhibit a decrease in peak intensity in the gel pore range, an increase in peak intensity in the macropore/capillary pore range, and little change in the void range. This suggests that after 3D printing, the number of gel pores decreases, while the number of capillary pores increases, with the quantity of voids remaining relatively constant. The increased number of capillary pores is a key factor contributing to the lower thermal conductivity of the printed specimens in the F0.0% group compared to the cast specimens.

For the SF1.0% group, the primary peaks in the pore size distribution curve of the cast specimen appear at 26.3 nm, 550.8 nm, and 144,531.8 nm, while those of the printed specimen shift to 23.4 nm, 1594.7 nm, and 179,467.7 nm. Similarly, for the PCF1.0% group, the primary peaks for the cast specimen are at 23.4 nm, 829.2 nm, and 177,747.4 nm, whereas those for the printed specimen shift to 21.1 nm, 1309.1 nm, and 179,567.7 nm. This indicates that compared to cast specimens, the primary peaks corresponding to capillary pores and voids in the pore size distribution curves of fiber-reinforced 3DPC-printed specimens shift toward larger pore sizes. This phenomenon suggests that the printing process leads to pore structure degradation, reducing the number of gel pores while increasing the number of capillary pores and voids.

For fiber-reinforced cast specimens, the pores are mainly concentrated below 50 nm, predominantly comprising gel pores. However, for fiber-reinforced printed specimens, the pore sizes range from 50 nm to 10 μm, with a higher proportion of macropores. This indicates that the presence of larger pores in fiber-reinforced 3DPC-printed specimens not only reduces their strength but also forms pathways for heat transfer, which is the primary reason why the thermal conductivity of fiber-reinforced 3DPC-printed specimens is higher than that of cast specimens.

#### 3.3.2. Influence of Fiber Surface Characteristics on Dispersion

Fiber surface characteristics can affect fiber–fiber interactions and consequently influence the agglomeration and dispersion behavior of fibers in cementitious matrices. In our previous study [19], microscopic observations showed that paraffin was predominantly stored within the internal pores of sisal fibers, while a smaller amount was retained on the fiber surface. This surface paraffin may modify contacts between adjacent fibers and their interactions with the surrounding aqueous phase. Pan et al. [57] used surfactants and paraffin wax to coat microcrystalline cellulose with the aim of limiting agglomeration during dispersion. Gustafsson et al. [58] showed that treatment with polyelectrolytes and paraffin wax colloids increased the hydrophobicity of cellulose fiber networks. These studies indicate that wax-containing surface treatments can modify the interfacial behavior of cellulose-based materials, although their treatment conditions differ from those used in the present study.

**Figure 18:**
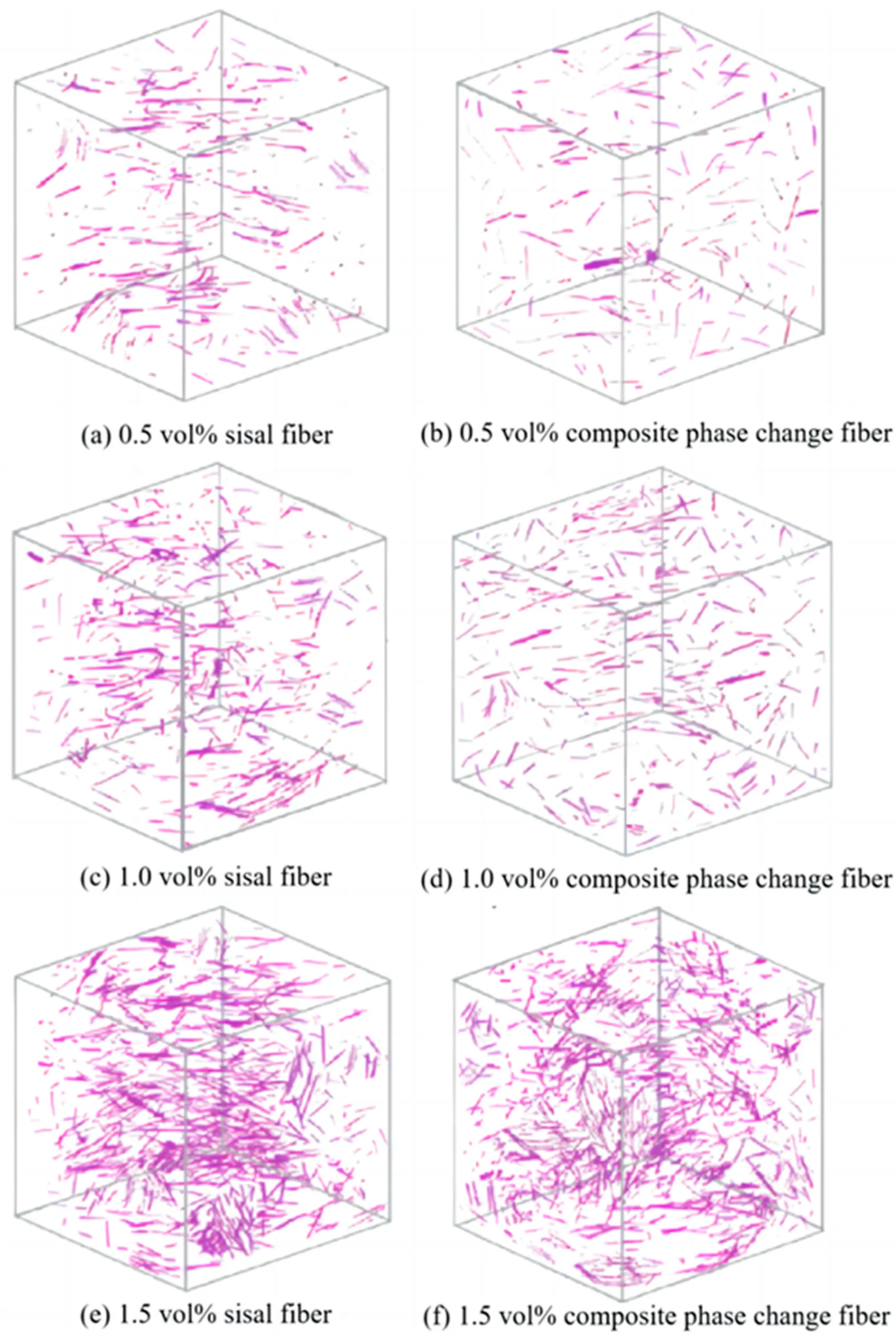
Three-dimensional reconstructed fiber distributions in cast specimens with different fiber volume fractions: (**a**) SF0.5%; (**b**) PCF0.5%; (**c**) SF1.0%; (**d**) PCF1.0%; (**e**) SF1.5%; and (**f**) PCF1.5%.

**Figure 19:**
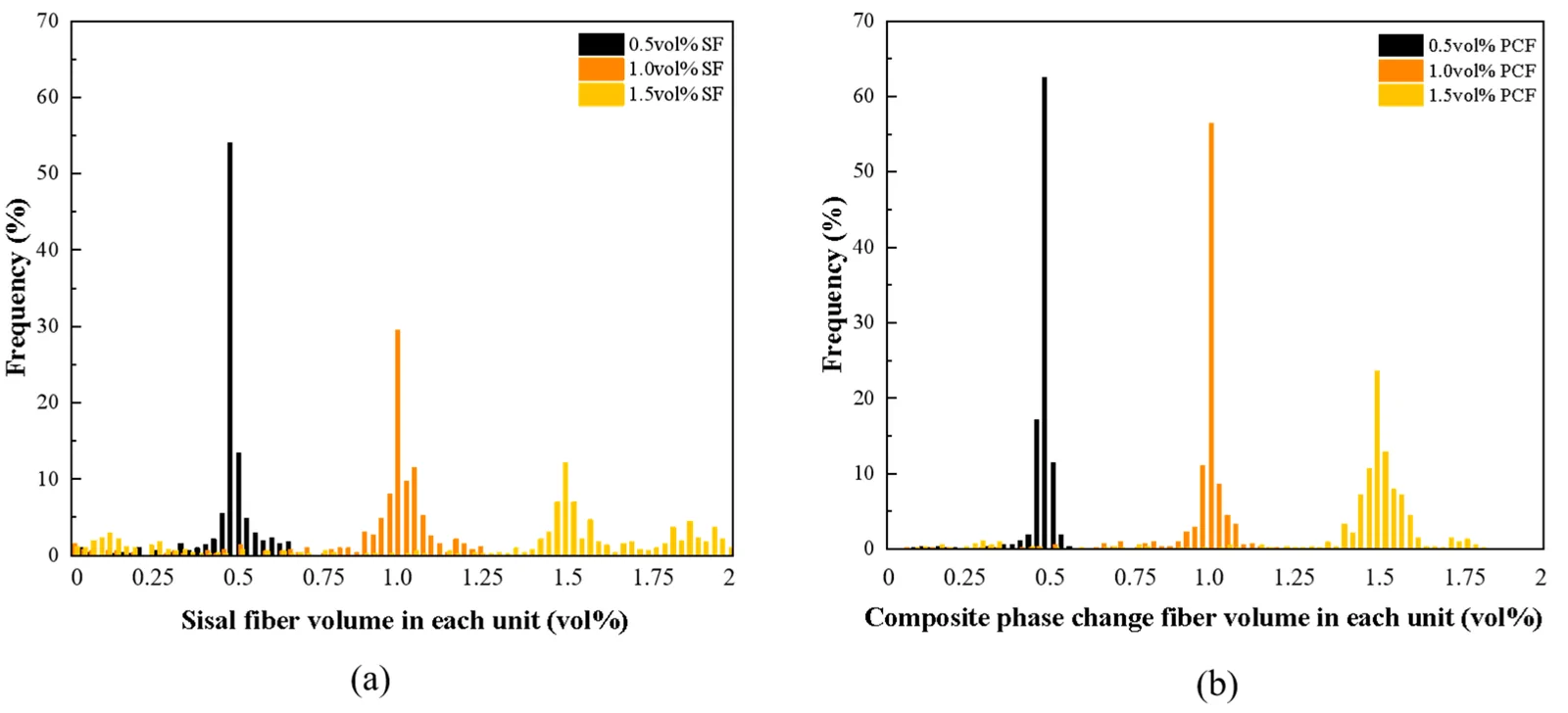
Dispersion degree of fibers for cast specimens: (**a**) sisal fibers; (**b**) phase change fibers.

Accordingly, paraffin retained on the phase change fiber surface may contribute to the lower agglomeration tendency inferred from the more uniform CT-based fiber distribution in Section 3.3.3. At the same fiber dosage, the phase change fibers exhibited a more uniform spatial distribution than the sisal fibers, as shown in Figure 18 and Figure 19.

#### 3.3.3. Fiber Spatial Distribution

The spatial distribution of the fibers, including their dispersion uniformity and orientation within the matrix, may contribute to the directional thermal properties of 3DPC. Fibers with a lower thermal conductivity than the surrounding matrix can modify local heat transfer, and their arrangement can also influence the effective thermal resistance of the composite. The CT observations indicate that the fibers in the printed specimens preferentially align along the printing direction. Such alignment, together with the pore structure and the printing interfaces, may underlie the differences in thermal resistance among the X, Y, and Z directions. For specimens of identical dimensions, the lower thermal conductivity measured along the X direction corresponds directly to a higher effective thermal resistance. This higher resistance may also partly account for the slower surface temperature rise observed along this direction, although the contribution of fiber orientation alone cannot be isolated from the present measurements.

Following the CT image processing and deep learning segmentation described in Section 2.3.5, the reconstructed fiber distributions in the cast specimens at different fiber dosages were obtained, as shown in Figure 18.

The image in Figure 18 was processed to analyze the distribution of fibers within the matrix. Following the procedure illustrated in Figure 7, the reconstructed volume of each specimen was divided into 512 unit cells of 5 × 5 × 5 mm^3^, and the fiber volume fraction was calculated for each unit cell. The dispersion uniformity of fibers in cast specimens was evaluated based on the variability of the fiber volume fraction, with the results shown in Figure 19.

From Figure 19, it can be observed that at the same fiber dosage, the probability of unit cells with the nominal fiber content appearing in the phase change fiber group is much higher than that in the sisal fiber group. This indicates that the phase change fibers exhibit better dispersion than the sisal fibers, which is consistent with the possible influence of paraffin-modified fiber surface characteristics discussed in Section 3.3.2.

**Figure 20:**
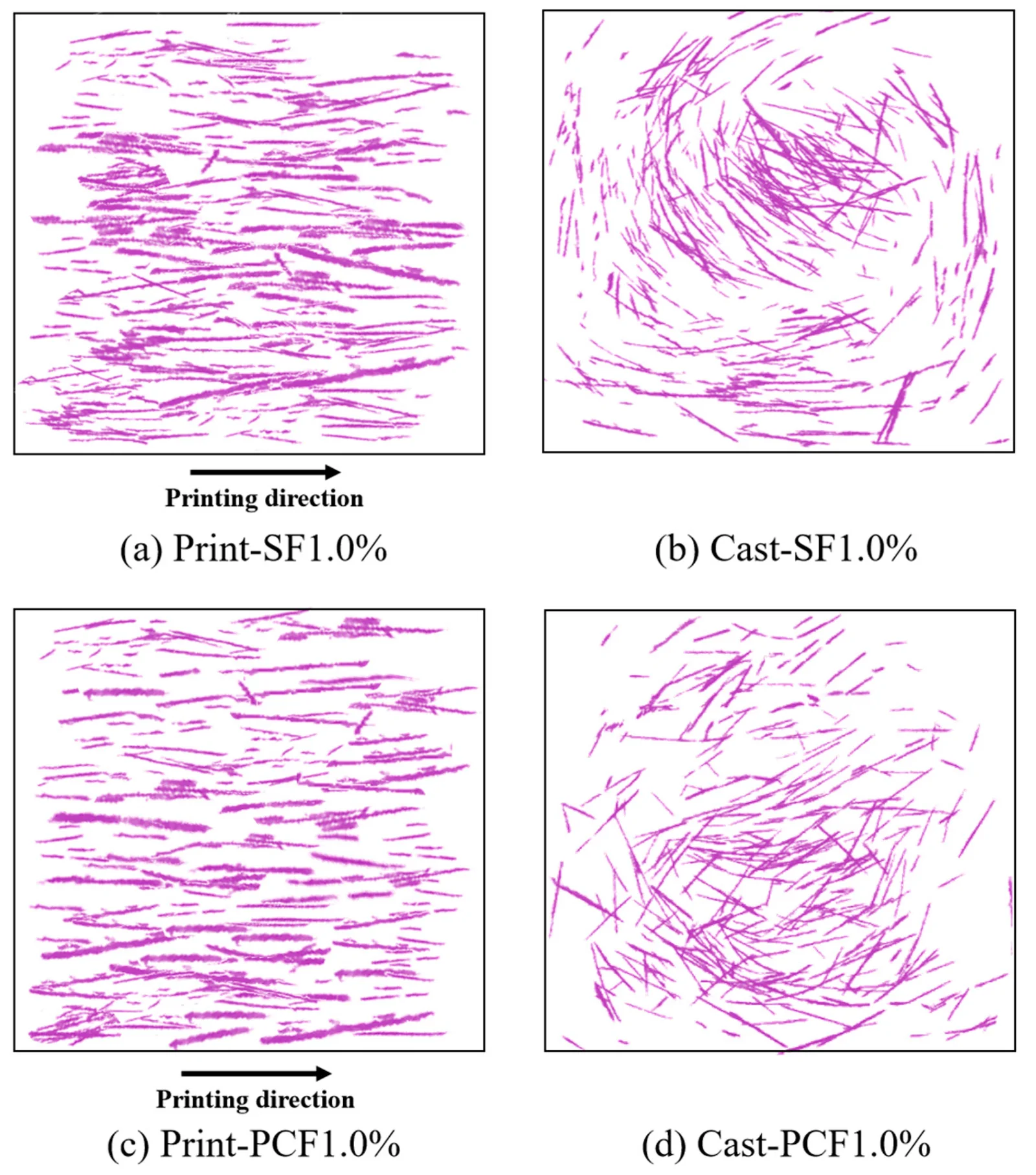
Fiber distribution in the cement-based composite specimens: (**a**) sisal fibers, printed specimen; (**b**) sisal fibers, cast specimen; (**c**) phase change fibers, printed specimen; (**d**) phase change fibers, cast specimen.

The three-dimensional reconstruction of fiber distribution in SF1.0% and PCF1.0% fiber-reinforced 3DPC-printed and cast specimens was performed, and the results are shown in Figure 20.

**Figure 21:**
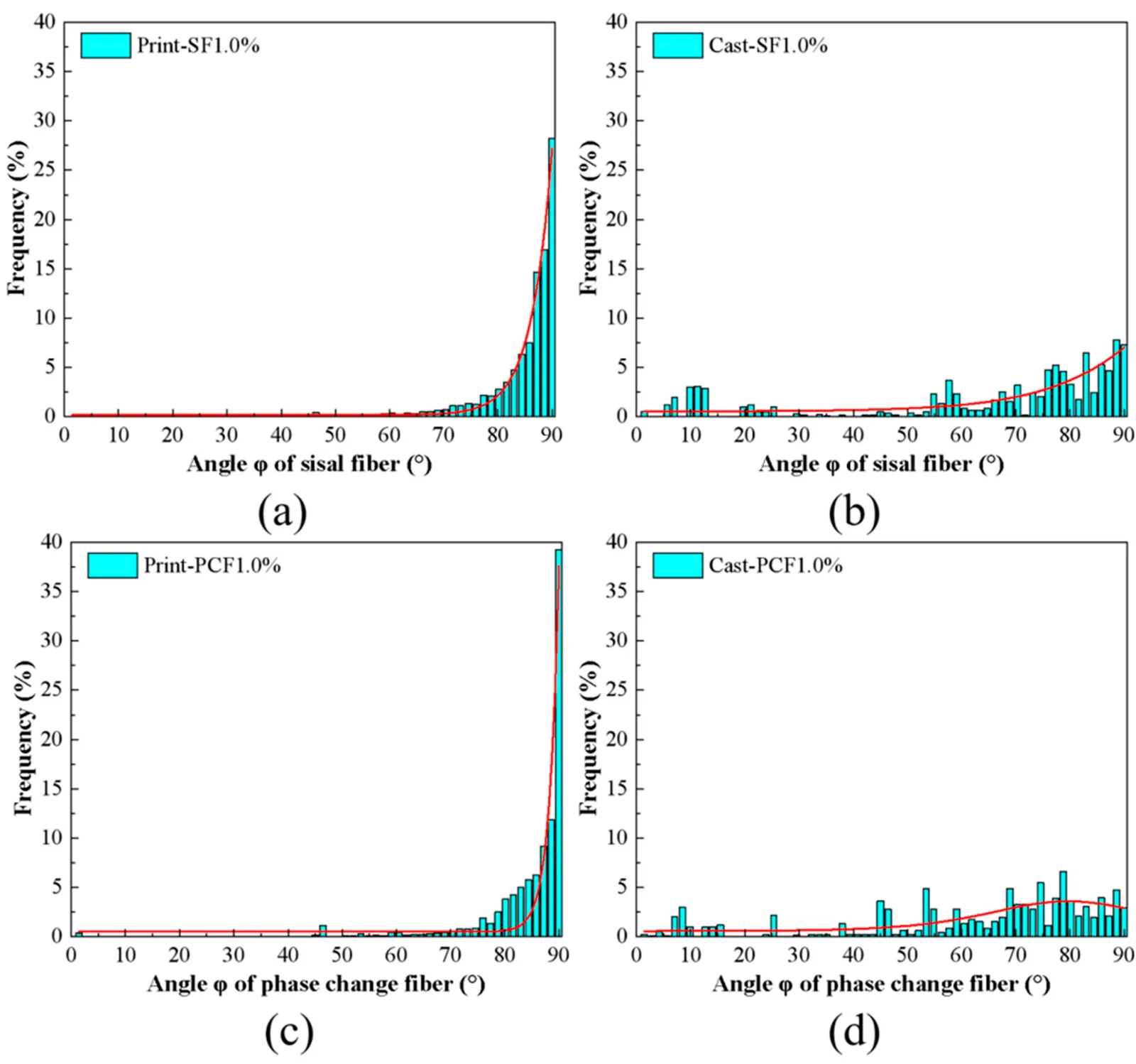
(**a**) Angle α distributions of sisal fiber in cement-based composite printed specimens; (**b**) angle α distributions of sisal fiber in cement-based composite cast specimens; (**c**) angle α distributions of phase change fiber in cement-based composite printed specimens; (**d**) angle α distributions of phase change fiber in cement-based composite cast specimens.

From Figure 20a,c, it can be observed that the fiber orientation in the printed specimens is parallel to the printing direction. From Figure 20b,d, it can be seen that the fibers in the cast specimens are more randomly distributed than those in the printed specimens, interweaving to form a spatial network structure while locally exhibiting an approximately concentric arrangement. The fiber orientation angle, denoted as α, was used to quantify the fiber spatial arrangement, and the statistical results were plotted in the form of a fiber orientation angle α histogram, as shown in Figure 21.

From Figure 21, it can be observed that the histogram for the printed specimens is shifted to the right compared to the cast specimens, indicating that most fibers in the printed specimens are aligned along the printing direction. When the fiber content is 1.0%, the α values in the printed specimens are concentrated in the range of 70° to 90°, whereas in the cast specimens, α values are distributed across the entire range of 0° to 90°. Similar experimental results were also reported by Pham [12].

The distribution of fibers within the matrix is influenced by multiple factors, including fiber type, fiber volume content, mix proportions, mixing process, and formwork wall effects [59]. The approximately concentric arrangement observed in the cast specimens may be associated with flow-induced fiber reorientation during mold filling. Previous studies have shown that radial spreading of fresh cementitious mixtures can promote fiber alignment transverse to the radial flow direction, thereby favoring a circumferential arrangement [60,61]. Mold boundaries may further modify local fiber orientations [62]. For printed specimens, the extrusion process through the nozzle plays a crucial role in fiber distribution [63]. From Figure 21a,c, it can be seen that in the printed specimens, the probability of fibers having a 90° orientation angle is 39.3% for phase change fibers and 28.2% for sisal fibers. This indicates that the alignment phenomenon of phase change fibers in printed specimens is more pronounced, further confirming that phase change fibers exhibit better dispersion.

## 4. Conclusions

This study investigated the effects of fiber type and dosage on the rheological and thermal properties of 3DPC and explained these effects from the perspectives of pore distribution and fiber dispersion. The following conclusions can be drawn:

(1) Phase change fibers exhibit better dispersion in the cementitious matrix than sisal fibers, which may be partially associated with changes in fiber–fiber contacts and fiber–water interactions caused by paraffin retained on the fiber surfaces. At the same fiber dosage, the initial static yield stress of 3DPC containing phase change fibers is significantly higher than that of 3DPC containing sisal fibers.
(2) At the same fiber dosage, 3DPC containing phase change fibers exhibited lower thermal conductivity and better thermal insulation performance along the X, Y, and Z directions than sisal-fiber-reinforced and fiber-free 3DPC. Phase change fibers might help reduce the heating/cooling loads and energy consumption of 3D-printed buildings, which remains to be verified.
(3) The mechanism by which phase change fibers enhance the rheological and thermal properties of 3DPC can be explained from multiple perspectives, including pore size distribution, fiber dispersion, and the intrinsic properties of the fibers. The more uniform fiber distribution observed by CT may contribute to the higher initial static yield stress of PCF-reinforced 3DPC, alongside differences in fiber water absorption and the corresponding mixing-water adjustments. Moreover, the incorporation of PCFs increases the capillary pores within the 3DPC, thereby reducing its effective thermal conductivity.

## Figures and Tables

**Table 1:** Chemical compositions of cement and silica fume by X-ray fluorescence (XRF) (%).

	CaO	SiO_2_	Al_2_O_3_	Fe_2_O_3_	MgO	SO_3_	K_2_O	Na_2_O	P_2_O_5_	TiO_2_	LOI
Cement	62.97	16.83	4.54	3.46	3.07	1.29	0.88	0.21	0.07	0.25	6.43
Silica fume	-	93.21	0.27	0.07	0.13	0.58	0.23	0.41	0.04	0.02	1.86

**Table 2:** Mix proportions of 3DPC (kg/m^3^).

Notation	Cement	Silica Fume	Sand	Water	HPMC	PCE	Fiber
F0.0%	697.4	60.6	1213	227.4	0.4	7.5	0
SF0.5%	697.4	60.6	1213	234.8	0.4	7.5	7.35
SF1.0%	697.4	60.6	1213	242.2	0.4	7.5	14.7
SF1.5%	697.4	60.6	1213	249.4	0.4	7.5	22.1
PCF0.5%	697.4	60.6	1213	232.1	0.4	7.5	7.35
PCF1.0%	697.4	60.6	1213	236.8	0.4	7.5	14.7
PCF1.5%	697.4	60.6	1213	241.5	0.4	7.5	22.1

## Data Availability

The original contributions presented in this study are included in the article. Further inquiries can be directed to the corresponding author.
